# Hepatic stellate cell-expressed endosialin balances fibrogenesis and hepatocyte proliferation during liver damage

**DOI:** 10.15252/emmm.201404246

**Published:** 2015-02-13

**Authors:** Carolin Mogler, Matthias Wieland, Courtney König, Junhao Hu, Anja Runge, Claudia Korn, Eva Besemfelder, Katja Breitkopf-Heinlein, Dorde Komljenovic, Steven Dooley, Peter Schirmacher, Thomas Longerich, Hellmut G Augustin

**Affiliations:** 1 Division of Vascular Oncology and Metastasis, German Cancer Research Center Heidelberg (DKFZ-ZMBH Alliance)Heidelberg, Germany; 2 Institute of Pathology, Heidelberg UniversityHeidelberg, Germany; 3 Department of Vascular Biology and Tumor Angiogenesis (CBTM), Medical Faculty Mannheim, Heidelberg UniversityHeidelberg, Germany; 4 Department of Medicine II, Section Molecular Hepatology - Alcohol Associated Diseases, Medical Faculty Mannheim, Heidelberg UniversityHeidelberg, Germany; 5 Division of Medical Physics in Radiology, German Cancer Research Center HeidelbergHeidelberg, Germany; 6 German Cancer ConsortiumHeidelberg, Germany

**Keywords:** angiocrine signaling, endosialin, liver fibrosis, liver regeneration

## Abstract

Liver fibrosis is a reversible wound-healing response to injury reflecting the critical balance between liver repair and scar formation. Chronic damage leads to progressive substitution of liver parenchyma by scar tissue and ultimately results in liver cirrhosis. Stromal cells (hepatic stellate cells [HSC] and endothelial cells) have been proposed to control the balance between liver fibrosis and regeneration. Here, we show that endosialin, a C-type lectin, expressed in the liver exclusively by HSC and portal fibroblasts, is upregulated in liver fibrosis in mouse and man. Chronic chemically induced liver damage resulted in reduced fibrosis and enhanced hepatocyte proliferation in endosialin-deficient (EN^KO^) mice. Correspondingly, acute-liver-damage-induced hepatocyte proliferation (partial hepatectomy) was increased in EN^KO^ mice. A candidate-based screen of known regulators of hepatocyte proliferation identified insulin-like growth factor 2 (IGF2) as selectively endosialin-dependent hepatocyte mitogen. Collectively, the study establishes a critical role of HSC in the reciprocal regulation of fibrogenesis vs. hepatocyte proliferation and identifies endosialin as a therapeutic target in non-neoplastic settings.

## Introduction

Nutritive–toxic, metabolic, or infectious challenges result in acute or chronic liver damage, affecting millions of patients worldwide (Poynard *et al*, 2010). Chronic liver damage leads to liver fibrosis (Lee & Friedman, 2011) that may progress to cirrhosis (Rahimi & Rockey, 2012), which is a major risk factor for hepatocellular carcinoma (HCC) (Rahimi & Rockey, 2011). A hallmark of liver fibrosis is the proliferation and transdifferentiation of hepatic stellate cells (HSC), which also serve as major producer of fibrogenic extracellular matrix (Hernandez-Gea & Friedman, 2011). Targeting HSC activation is therefore considered a promising approach to reduce liver fibrosis and the risk of cirrhosis (Ebrahimkhani *et al*, 2011; Inagaki *et al*, 2012). HSC also affects hepatocyte proliferation through paracrine signaling mechanisms and thereby contributes to liver regeneration (Ebrahimkhani *et al*, 2011). Similarly, endothelial cells control liver regeneration (Ding *et al*, 2010; Hu *et al*, 2014) and the concepts of endothelial angiocrine signaling have recently been expanded to liver fibrosis (Ding *et al*, 2014). Thus, liver fibrosis and hepatocyte proliferation appear to be reciprocally regulated processes.

The C-type lectin-like transmembrane protein endosialin was originally identified as marker of angiogenic endothelial cells (Rettig *et al*, 1992; St Croix *et al*, 2000; Christian *et al*, 2001). However, later work by us and others revealed that endosialin is not a marker of angiogenic endothelial cells, but rather a marker of activated pericytes and tumor stromal myofibroblasts (Rettig *et al*, 1992; St Croix *et al*, 2000; MacFadyen *et al*, 2005, 2007; Christian *et al*, 2008; Simonavicius *et al*, 2008). Based on its strict oncofetal expression, endosialin has consequently been proposed as a tumor stroma therapeutic target. In fact, clinical trials with an endosialin antibody (MORAb-004) are ongoing (www.clinicaltrials.gov/ct2/results?term=endosialin&Search=Search). Likewise, endosialin has been proposed as a target for tumor immune-PET applications (Chacko *et al*, 2014) and for tumor vasculature targeting vaccination strategies (Facciponte *et al*, 2014).

Based on the exclusive expression of endosialin by activated pericytes and tumor stromal myofibroblasts and also stimulated by ongoing efforts to translate endosialin as a therapeutic target, we hypothesized that endosialin may be a marker of activated hepatic stellate cells, which are the organ-specifically specialized pericytes of the liver. This study was consequently aimed at analyzing the role of endosialin during liver fibrosis and liver regeneration.

## Results and Discussion

### Upregulation of endosialin expression by activated hepatic stellate cells in early liver fibrogenesis

**Figure 1:**
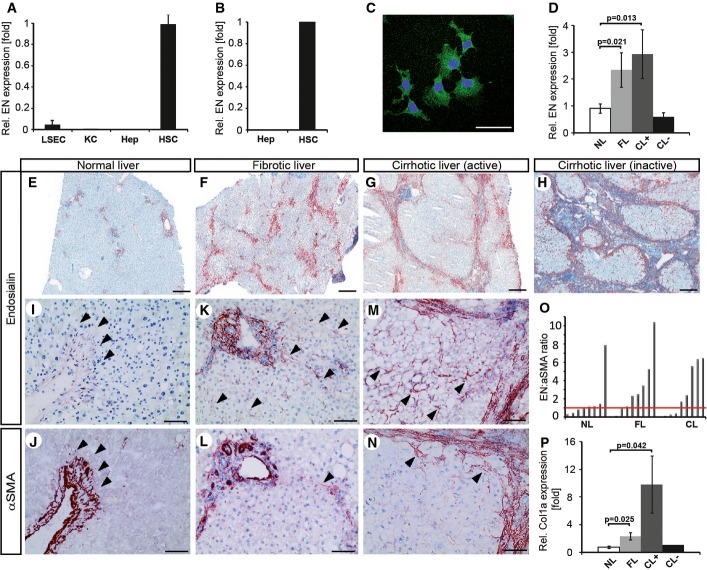
Upregulation of endosialin (EN) expression by activated hepatic stellate cells (HSC) in early liver fibrogenesis
A EN qRT–PCR of murine liver sinusoidal endothelial cells (LSEC), Kupffer cells (KC), hepatocytes (Hep), and HSC (*n* = 3 per cell type).B EN qRT–PCR of human hepatocytes (*n* = 1) and HSC cell line LX-2 (*n* = 3).C EN immunofluorescence staining of LX-2 cells. Scale bar: 25 μm.D EN qRT–PCR of normal liver (NL) and fibrotic liver (FL,*n* = 8 each), and active or inactive cirrhotic liver (CL+/−, *n* = 10/ *n* = 2).E-H EN immunohistochemistry staining of human normal liver (E), fibrotic liver (F), and cirrhotic liver (G, H). Weak EN expression in portal tracts of normal liver (E). Abundant EN expression detected in activated HSC in fibrous septa of fibrotic (F), active cirrhotic (G), and inactive cirrhotic liver (H). Scale bars: 500 μm.I-N Higher magnification of EN-stained normal liver (I), fibrotic liver (K), and (active) cirrhotic liver (M) compared to alpha-smooth muscle actin (αSMA) immunohistochemistry on serial slides (J, L and N). Scale bars: 100 μm.O EN/αSMA mRNA ratio in normal liver, fibrotic liver, and active cirrhotic liver.P Collagen 1a (Col1a) expression in normal, fibrotic, and active cirrhotic liver determined by qRT–PCR.
Data information: Data are expressed as mean ± s.e.m.; *P*-value (significant < 0.05) determined by Student's *t*-test.

Corresponding to the reported expression of EN by activated mesenchymal cells including pericytes and (myo)fibroblasts (Christian *et al*, 2008), weak expression restricted to HSC and portal fibroblasts was detected in healthy adult murine and human livers (Fig1A–C and E). EN was strongly upregulated in human liver fibrosis (FL) and active cirrhosis (CL+) showing immunhistochemical overlap with well-established pericyte and myofibroblast markers (such as PDGFR beta or α-smooth muscle actin) but never with endothelial markers (Fig1D, F and G; Supplementary Figs S1 and S2). Downregulated EN expression during late-stage cirrhosis (Fig1D and H) identified EN as an early HSC activation marker. Comparative analysis of EN expression with the established HSC activation marker α-smooth muscle actin (αSMA) identified a substantially stronger differential of EN expression in diseased vs. healthy tissue (Fig1I and M versus J and N). Correspondingly, qRT–PCR analyses determined a significant change in the ratio of EN-to-αSMA mRNA expression comparing normal with fibrotic or cirrhotic liver (Fig1O). EN expression was also more pronounced in early stages of fibrogenesis compared to collagen 1a, the main product of HSC in fibrotic tissue (Fig1P).

### Reduced fibrosis in EN-deficient mice in chronic liver damage

**Figure 2:**
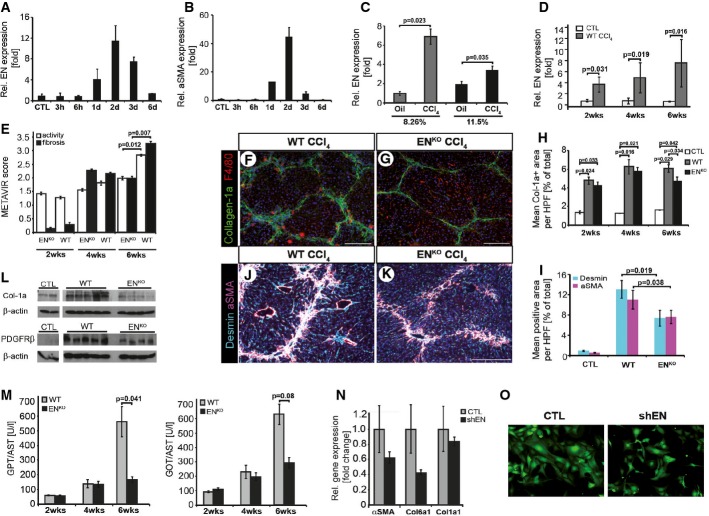
Reduced fibrosis in EN-deficient mice (EN^KO^) in chronic liver damage
A, B qRT–PCR for EN (A) and αSMA (B) of liver lysates from single high-dose CCl_4_- or oil-treated wild-type (WT) mice (*n* = 2 per time point).C Early activated and long-term-activated HSC isolated by differential density gradient centrifugation (8.26% versus 11.5% density layer). qRT–PCR for EN from early activated (8.26%) and long-term-activated HSC (11.5%) from 4-week CCL_4_-treated mice and vehicle control mice (*n* = 3–4 per data point).D EN expression in liver lysates from 2-, 4-, and 6-week CCl_4_-treated mice (*n* = 5–8) and vehicle control mice (*n* = 3) determined by qRT–PCR.E-G METAVIR score of 2-, 4-, and 6-week CCl_4_-treated mice (F–G) Col1a and F4/80 immunofluorescence of 6-week CCl_4_-treated and vehicle control WT (F) and EN^KO^ (G) mice.H Mean Col1a^+^ area per high power field (HPF) shown as percentage of the total area (*n* = 5–8).I Western blot analysis for collagen 1a (Col1a), platelet-derived growth factor receptor beta (PDGFRβ) and β-actin of total liver protein lysates from 6-week CCl_4_-treated or control vehicle WT and EN^KO^ mice (*n* = 3–5).J-L Double immunofluorescence of 6-week CCl_4_-treated and vehicle control WT (J) and EN^KO^ (K) mice for desmin and αSMA (L) shown as percentage of the total area (*n* = 5–8).M Serum liver enzymes (GPT/ALT and GOT/AST) from 2-, 4-, and 6-week CCl_4_-treated WT and EN^KO^ mice (*n* = 5–8).N qRT–PCR of endosialin (EN), αSMA, and collagen 1a1/6a1 from lentiviral-mediated endosialin knockdown (shEN) and control-transfected (CTL) human hepatic stellate cells (LX-2).O Lentiviral-mediated endosialin knockdown (shEN) and control (CTL) in LX-2 cells (*n* = 3). Magnification 10×.
Data information: Scale bars: 250 μm. CTL = vehicle control mice. Data are expressed as mean ± s.d. (A–D), or s.e.m. (E, H, L and M); *P*-value (significant < 0.05) determined by Student's *t*-test. Source data are available online for this figure.

To study the consequences of acute liver damage *in vivo*, hepatotoxicity was induced in mice by injection of a single dose of carbon tetrachloride (CCl_4_). Acute liver toxicity led to rapid upregulation of EN and αSMA (Fig2A and B), followed by upregulated collagen 1a expression (Supplementary Fig S3). Analysis of isolated cell populations identified the vitamin A+, early activated HSC population (8.26% density gradient layer) as the primary EN-positive cell population (Fig2C).

**Figure 3:**
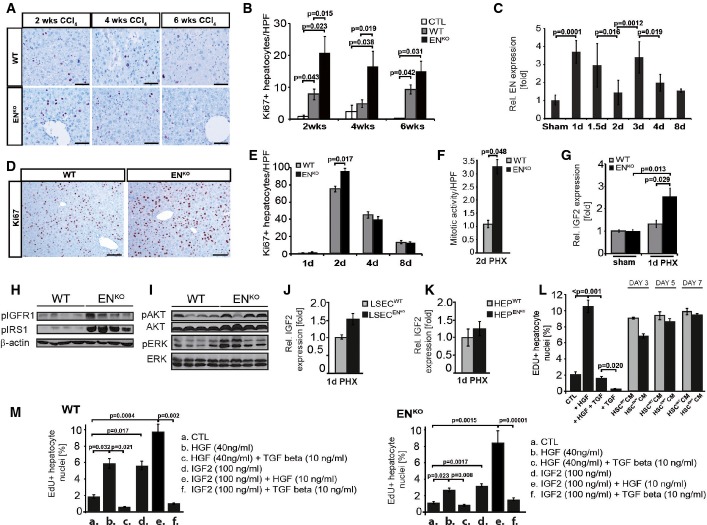
Negative regulation of hepatocyte proliferation by HSC-expressed endosialin
A Representative images of Ki-67 immunohistochemistry after 2, 4, and 6 weeks of CCl_4_ treatment of WT and EN^KO^ mice (*n* = 5–8 mice per group and time point). Scale bars: 75 μm.B Quantitation of Ki67^+^ hepatocytes/HPF after 2, 4, and 6 weeks of CCl_4_ treatment.C Time course of EN expression after partial hepatectomy (2/3 PHx) determined by qRT–PCR of total liver lysates (*n* = 5/time point, sham: *n* = 3).D Representative images of Ki-67 immunohistochemistry 2 days after 2/3 PHx of WT and EN^KO^ mice (*n* = 5–9 mice per group and time point).E Quantitation of Ki-67^+^ hepatocytes/HPF determined 1, 2, 4, and 8 days after 2/3 PHx.F Number of mitotic figures 2 days after 2/3 PHx of WT and EN^KO^ mice (*n* = 5–9 mice per group and time point).G qRT–PCR analysis of total liver lysates 1 day after PHx for IGF2.H Western blot analysis of phosphorylated insulin-like growth factor receptor 1 (pIGFR1), phosphorylated insulin receptor substrate 1 (pIRS1) and β-actin 2 days after PHx.I Western blot analysis of phosphorylated and total extracellular signal-regulated kinase (ERK) and AKT 2 days after PHx.J, K qRT–PCR of IGF2 in isolated liver sinusoidal endothelial cells (LSEC)(J) or hepatocytes (HEP)(K) 1 day after PHx.L EdU-positive hepatocytes (in %) after 24-h stimulation with conditioned medium (CM) from either WT or EN^KO^ HSC (3, 5, or 7 days of activation). HGF (40 ng/ml) and TGF-β (10 ng/ml) serve as positive or negative control, respectively.M EdU-positive WT and EN^KO^ hepatocytes (in %) after stimulation with IGF2 (100 ng/ml). HGF (40 or 10 ng/ml) and TGF-β (10 ng/ml) serve as positive or negative control, respectively.
Data information: Scale bars (D, E): 100 μm. Data are expressed as mean ± s.e.m. *P*-value (significant < 0.05) determined by Student's *t*-test. Source data are available online for this figure.

Based on the observed prominent regulation of EN in a mouse model of acute liver damage, we next performed comparative hepatotoxicity experiments in wild-type (WT) and EN-deficient mice (EN^KO^). EN^KO^ mice are viable and fertile and display no overt phenotype unless they are pathologically challenged (Nanda *et al*, 2006). Likewise, comparative analyses of WT and EN^KO^ livers from adult mice showed no overt morphological and liver-related clinical chemistry alterations (Supplementary Fig S4). A single high-dose injection of CCl_4_ led to an equal extent of liver damage with acute zonal necrosis and increase in serum liver enzymes in both genotypes (Supplementary Fig S5). Twice weekly chronic CCl_4_ administration resulted in a continuous increase in EN expression in WT mice over a period of 6 weeks (Fig2D). Overall liver damage (assessed by cleaved caspase-3 activity) was similar in WT and EN^KO^ mice (Supplementary Fig S6; Fig2F and G). Likewise, the initiation of liver fibrosis was comparable in WT and EN^KO^ mice as indicated by similar levels of αSMA and desmin expression (Supplementary Fig S7). Yet, fibrosis progression was delayed in EN^KO^ mice as evidenced by significantly ameliorated (necro-)inflammatory activity (assessed by METAVIR Score), reduced levels of collagen 1a, PDGFR beta, αSMA, desmin, and TIMP1 expression as well as lower liver serum enzymes after 6 weeks of repeated CCl_4_ administration (Fig2E–M; Supplementary Figs S8, S9 and S10). No differences in epithelial-to-mesenchymal transition were observed (Supplementary Fig S11). Fully activated hepatic stellate cells isolated from either wild-type or EN^KO^ mice showed similar cell morphology after 8 days in culture (Supplementary Fig S12). However, lentiviral-mediated endosialin knockdown in human immortalized hepatic stellate cells (LX-2 cells) revealed dramatically reduced levels of αSMA and collagens 1 and 6 (Fig2N) as well as a reduced myofibroblastic phenotype in the shEN HSC compared to control-transfected cells (Fig2O). While the initiation of liver fibrosis was not altered in EN^KO^ mice, hepatocyte proliferation was significantly increased in EN^KO^ mice compared to WT mice, most notably at the early time points (Fig3A and B, Supplementary Fig S13), resulting in an increased mean bodyweight after 6 weeks of CCl_4_ treatment (Supplementary Fig S14). These findings suggested that the observed ameliorated fibrosis phenotype after 6 weeks might not just have resulted from reduced fibrogenic activity, but rather a shift from a profibrogenic to a proregenerative phenotype in EN^KO^ mice as has previously been observed in 5-hydroxtryptamine 2B receptor (5-HT_2B_)-deficient mice (Ebrahimkhani *et al*, 2011).

### Negative regulation of hepatocyte proliferation by HSC-expressed endosialin

We next performed comparative partial hepatectomy (PHx) experiments in WT and EN^KO^ mice in order to examine the role of HSC-expressed EN in a model of rapid hepatocyte proliferation. PHx resulted in a characteristic biphasically upregulated expression of EN (Fig3C) in HSC with maximum EN expression 1 day after PHx (coinciding with the initiation of hepatocyte proliferation) and 3 days after PHx (coinciding with the initiation of stromal cell proliferation) (Michalopoulos & DeFrances, 1997; Miyaoka *et al*, 2012). Hepatocyte proliferation was significantly increased in EN^KO^ mice after 2/3 and 1/3 PHx (Fig3D and E; Supplementary Figs S15 and S16) showing more mitotic figures (Fig3F), but equal numbers of double nuclei (Supplementary Fig S17). EN^KO^ livers presented with smaller hepatocytes 2 days after hepatectomy, resulting in only slight differences in the liver-to-bodyweight ratio (Supplementary Fig S17). A candidate-based screen of known hepatocyte mitogens identified significantly upregulated levels of IGF2 in livers of EN^KO^ mice (Fig3G). IGF2 has been proposed to act as a regulator of hepatocyte proliferation (Kimura & Ogihara, 1998), which corresponds to earlier work showing that IGF signaling during liver regeneration affected hepatocyte turnover but not necessarily cell volume (Leu *et al*, 2003). IGF receptor 1 and IRS1 were activated in EN^KO^ mice. Likewise, phosphorylation of downstream signaling pathways such as ERK and AKT was upregulated in EN^KO^ mice (Fig3H and I). In turn, the expression of other hepatocyte mitogens such as IGF1, HGF, TGF-β1, TGF-β2, TGF-β3, HB-EGF, FGF21, IL-1β, TIMP, or Cdkn1b was not altered (Supplementary Figs S18 and S19). The elevated levels of IGF2 identified in whole liver lysates could not be attributed to a particular cell type in the liver since liver sinusoidal endothelial cells (Fig3J), hepatocytes (Fig3K), and HSC (Supplementary Fig S20) were all identified as source of IGF2. Endosialin did not appear to directly affect IGF2 production in HSC as EdU uptake of cultured hepatocytes stimulated with conditioned medium collected from variably activated wild-type or EN^KO^ HSC was increased to the same extent (Fig3L). To examine whether IGF2 was able to directly affect hepatocyte proliferation, isolated hepatocytes from both genotypes were stimulated with recombinant IGF2, leading to a significantly increased EdU uptake after 24 h of stimulation (Fig3M). Together, the selective regulation of IGF2 in livers from EN^KO^ mice suggests a possible role in the observed hepatocyte proliferation phenotype. Yet, further work will be needed to unravel the molecular details of the presumed endosialin–IGF2 axis.

In conclusion, this study has identified the hepatic stellate cell (HSC) marker endosialin (EN) as a critical balance of liver fibrogenesis and regenerative hepatocyte proliferation. Based on three independent pathology models (acute and chronic CCl_4_-induced liver damage, fibrosis, and partial hepatectomy) and validated by corresponding human pathology specimen expression profiling analyses, EN serves as positive regulator of the fibrogenic stromal cell compartment and as negative regulator of the parenchymal cell hepatocytic compartment (Supplementary Fig S21). Future work will need to unravel the molecular details of the paracrine endosialin-mediated cross talk between HSC and hepatocytes, most notably the role of IGF2 as a regulator of hepatocyte proliferation. Yet, the identification of an HSC-specifically expressed targetable and druggable cell surface receptor that controls the balance between liver fibrosis and liver regeneration may pave the way to fibrosis targeting and at the same time liver regeneration enhancing therapies: The dual compartment functions of endosialin further establish the molecular interdependency of HSC and hepatocyte contribution to liver function in health and disease (Ebrahimkhani *et al*, 2011). Moreover, EN targeting is presently pursued in early clinical tumor trials. Thus, the data identify EN as an attractive non-oncologic target for liver fibrosis, whose therapeutic inhibition could negatively impact fibrosis, while at the same time stimulate hepatocyte proliferation.

## Materials and Methods

### Patient samples

Tissue samples were provided by the tissue bank of the National Center for Tumor Diseases (NCT, Heidelberg, Germany) in accordance with the regulations of the tissue bank and the approval of the ethics committee of the University of Heidelberg. This study was performed according to the Declaration of Helsinki; written informed consent was obtained from all patients. All patient specimen and corresponding clinical information were exclusively provided in a pseudonymized form according to the standard operating procedures of the NCT, approved by the ethic committee of the University of Heidelberg (Ethikvotes #206/207, 2005). Primary human hepatocytes were kindly provided by PD Dr. K. Breuhahn (Institute of Pathology, Heidelberg).

### Animal experiments

All animal experiments were performed according to the guidelines of the local animal use and care committees and approved by the Regierungspräsidium in Karlsruhe, including animal permits G195/10 (CCl_4_ experiments) and G220/11 (partial hepatectomy). Animals were housed in barriers at the animal facility of the DKFZ with free admission to food and water. For sacrificing, mice were anesthetized by intraperitoneal injections of ketamine (100 mg/kg) and xylazine (10 mg/kg) diluted in isotonic 0.9% NaCl. Deeply anesthetized mice were heart punctured to obtain blood followed by rapid dislocation of the cervical spine. Whole blood was centrifuged at 8,000 *g* for 10 min, and subsequently, serum was collected from the supernatant. For determining clinically relevant serum parameters, 300 μl of serum (if necessary diluted with isotonic 0.9% NaCl) was analyzed by the Diagnostic Center of the Heidelberg University Hospital.

To induce acute liver damage, C57/Bl6 WT mice at an age of 10 weeks were injected intraperitoneally with a single high dose of CCl_4_ (1.6 g/kg CCl_4_, diluted in mineral oil). Mice were sacrificed after 3, 6, 24, 48, 72 h, or 6 days after injection. CCl_4_-induced liver fibrosis experiments were performed as previously described (Constandinou *et al*, 2005). Five to seven male C57/Bl6 endosialin WT and KO mice group at an age between 10 and 15 weeks were used for these experiments. Mice were injected intraperitoneally with CCl_4_ (1 ml/kg bodyweight, 1:7 dilution in olive oil) twice a week for 2, 4, or 6 weeks. Control mice received the same amount of olive oil as CCl_4_-treated animals during the experimental time. Mice were sacrificed 3 days after the last CCl_4_ injection. Partial hepatectomy was performed as previously described (Mitchell & Willenbring, 2008). In case of performing 1/3 partial hepatectomy, only left lobe was removed. Five to ten male C57BL/6 endosialin WT and KO mice group at an age between 10 and 15 weeks were used for partial hepatectomy experiments. Mice were anaesthetized by intraperitoneal injections of ketamine (100 mg/kg) and xylazine (10 mg/kg) diluted in isotonic 0.9% NaCl. As control, mice were sham-operated. Mice were sacrificed after 1, 1.5, 2, 4, or 8 days posthepatectomy.

### Statistical analysis

All results are expressed as mean ± s.e.m. or s.d., as indicated. The statistical differences between WT and EN^KO^ mice groups were analyzed using the two-tailed unpaired Student's *t*-test. The statistical difference between different time points in WT mice was analyzed using the two-tailed paired or unpaired *t*-test. Correlations were analyzed using the Pearson (bivariate) correlation. Differences *P* ≤ 0.05 were considered statistically significant.

### Additional materials and methods

Detailed information on additional materials and methods related to cells and cell culture experiments, molecular techniques, biochemical protocols and reagents, and histological and immunohistological techniques are summarized in the Supplementary Information.

## Supporting Information

Supplementary Information

Review Process File
