# Supplementary material for: Hepatic stellate cell-expressed endosialin balances fibrogenesis and hepatocyte proliferation during liver damage
Source: EMBO Mol Med. 2015 Feb 13;7(3):332–8. doi: 10.15252/emmm.201404246 (PMC4364949; doi:10.15252/emmm.201404246)
Supplement: Supplementary file 1 [file emmm0007-0332-sd1.pdf]

Online supplement

Hepatic stellate cell expressed Endosialin balances fibrogenesis and hepatocyte proliferation during liver damage

Carolin Mogler, Matthias Wieland, Courtney König, Junhao Hu, Anja Runge, Claudia Korn, Eva Besemfelder, Katja Breitkopf-Heinlein, Dorde Komljenovic, Steven Dooley, Peter Schirmacher, Thomas Longerich, Hellmut G. Augustin

Online supplement – Table of content

page

Suppl. Figure 1: Additonal myofibroblast and pericyte markers of human liver tissue 3

Suppl. Figure 2: Immunhistochemical ouble stains of Endosialin and CD31 4

Suppl. Figure 3: q rt PCR of collagen 1 after CCl_4 acute (high dose) treatment 5

Suppl. Figure 4: Histology and liver function tests of EN^{KO} mice 6

Suppl. Figure 5: Histology and liver function tests of CCL_4 acute treatment 7

Suppl. Figure 6: leaved aspase nd acrophage uantitation xperimental ibrosis 8

Suppl. Figure 7: αSMA and Desmin quantitation after 2 and 4 weeks of CCl_4 treatment 9

Suppl. Figure 8: Western blot f αSMA after 6 weeks of CCl_4 treatment 10

Suppl. Figure 9: q rt PCR of TIMP1 after 2,4 and 6 weeks of CCl_4 treatment 11

Suppl. Figure 10: Correlation of liver function tests and collagen levels after 6 weeks of CCl_4 treatment . 12

Suppl. Figure 11: q rt PCR of epithelial to mesenchymal transition markers 13

Suppl. Figure 12: Morphology of HSC activation in wildtype and EN^{KO} mice 14

Suppl. Figure 13: Additional proliferation markers in liver fibrosis 15

Suppl. Figure 14: Liver to body weight ratios in experimental liver fibrosis 16

Suppl. Figure 15: Hepatocyte proliferation in 1/3 partial hepatectomy 17

Suppl. Figure 16: Additional proliferation markers in 2/3 partial hepatectomy 18

Supplementary Figure 17: Histological evaluation and liver to bodyweight ratio in 2/3 hepatectomy . . 19

Supplementary Figure 18: q rt PCR of IGF1 two days after hepatectomy 20

Supplementary Figure 19: q rt PCR of established hepatocyte mitogens 21

Supplementary Figure 20: q rt PCR of IGF2 in HSC after 8 days of cultivation 22

Supplementary Figure 21: Conceptional model of Endosialin functionality in liver health and disease . . 23

Supplementary Material & Methods related to cells and cell culture experiments, molecular . . . . 24 techniques, biochemical protocols and reagents, as well as histological and immunohistological techniques.

Supplementary Figure 1
Mogler et al.

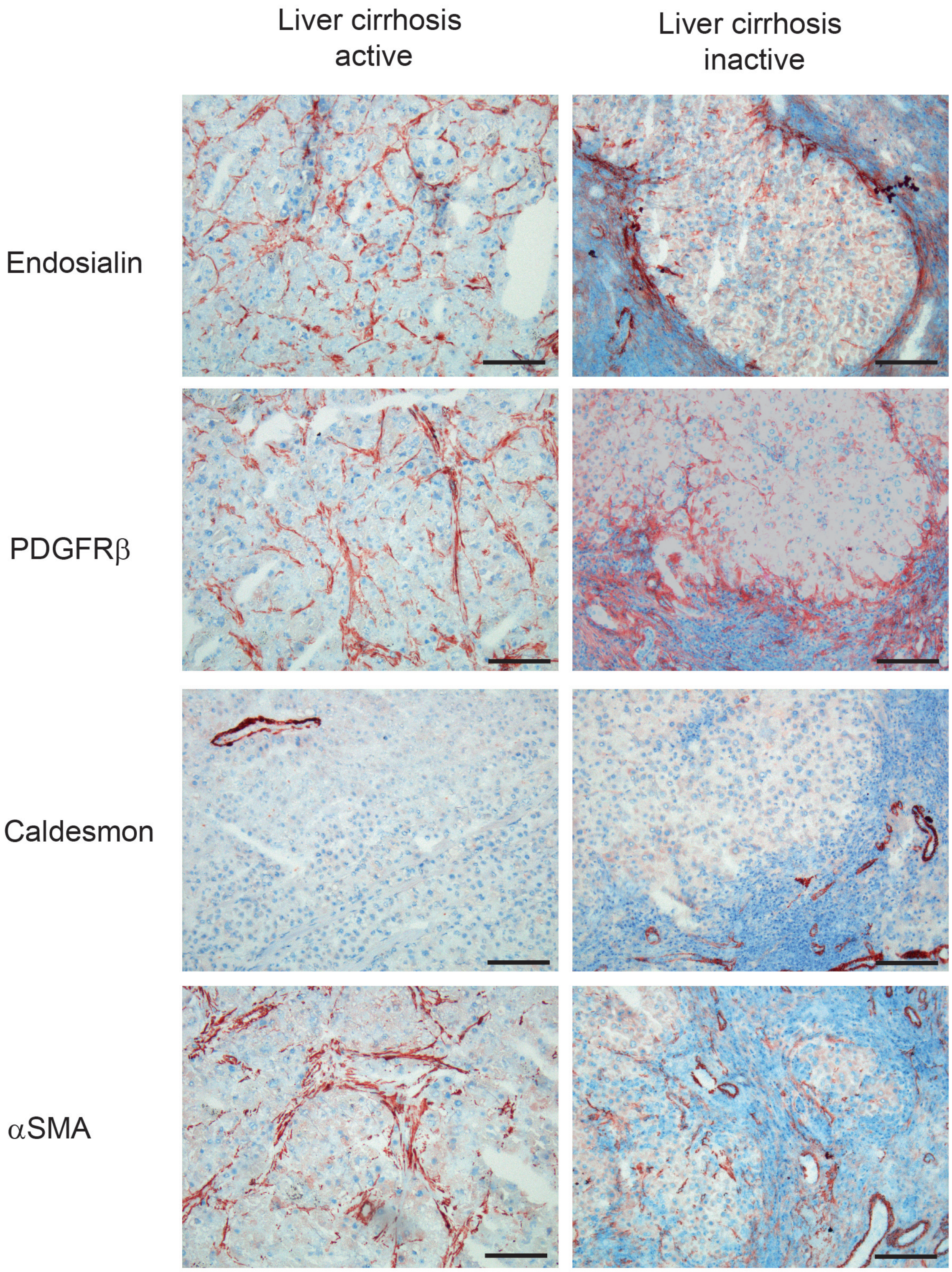

Immunohistochemical stainings of human liver cirrhosis samples (serial sections) against Endosialin, platelet derived growth factor receptor beta (PDGFRβ), caldesmon and alpha smooth muscle actin (αSMA). Scale bar: 100µm

Supplementary Figure 2
Mogler et al.

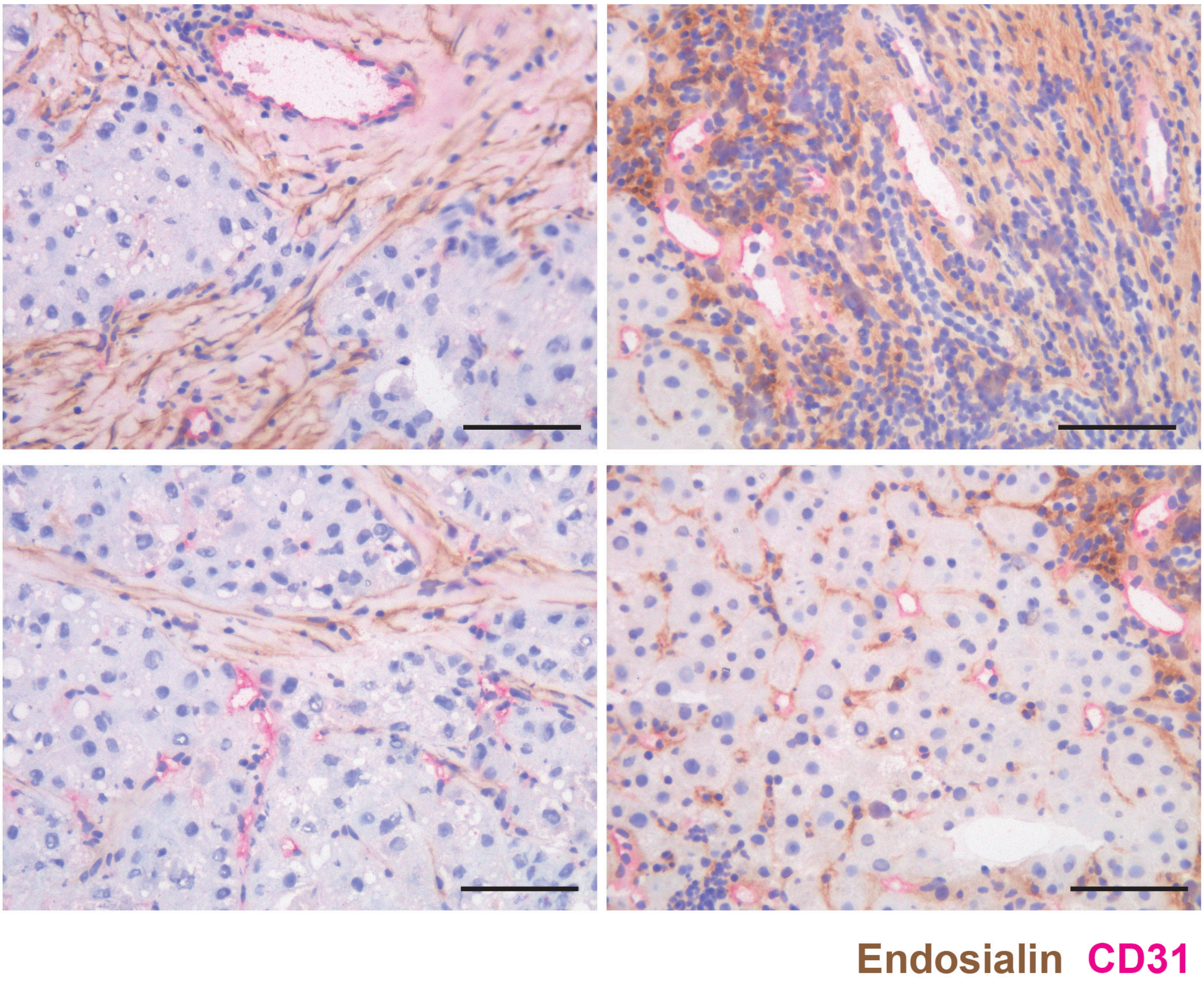

Immunohistochemical doublestainings of human liver cirrhosis samples against Endosialin (brown) and CD31 (red). Scale bar: 100µm

Supplementary Figure 3
Mogler et al.

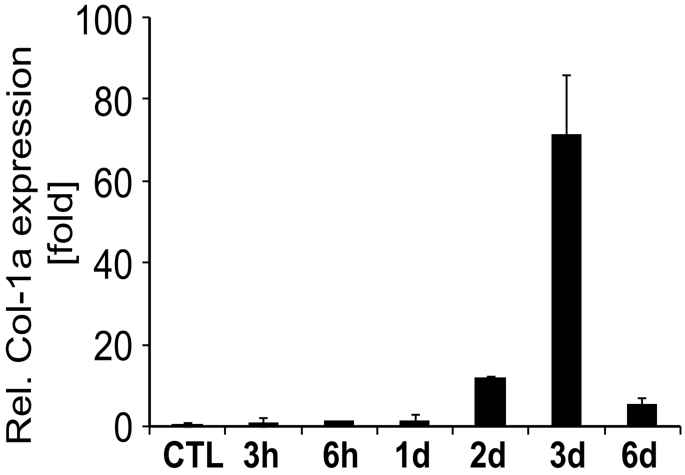

Q-RT-PCR for collagen 1a (Col1a) of total liver lysates from single high dose CCl_4- or oil-treated wild type (WT) mice (n=2 per time point).

Supplementary Figure 4
Mogler et al.

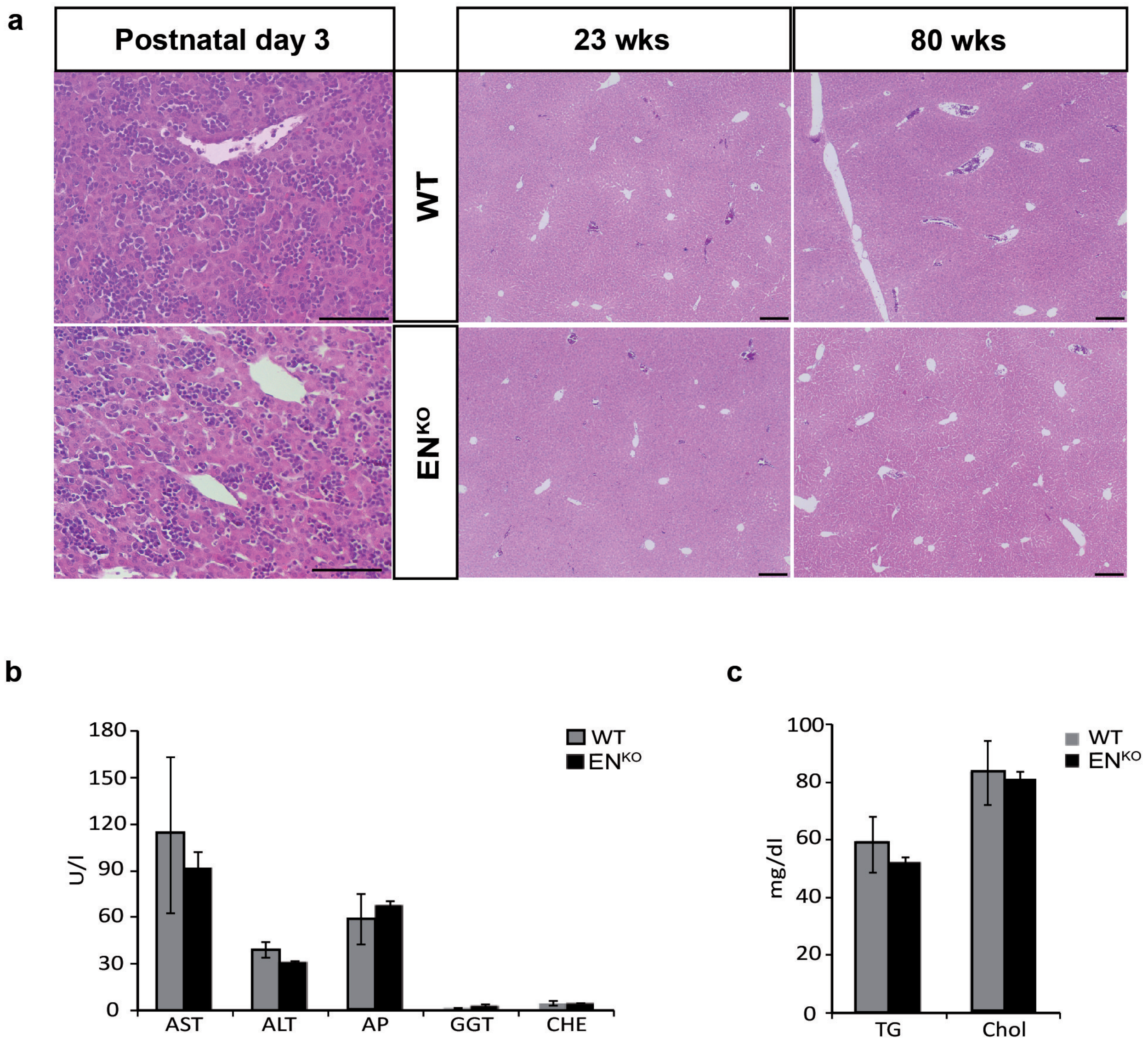

Healthy WT and EN^{KO} mice were sacrificed at different developmental stages. Livers were formalin fixed, paraffin embedded followed and stained for HE s. **(a)** No difference was observed between WT and EN^{KO} mice at neither age. **(b-c)** Blood was taken by heart puncture of anesthetized mice. Clinical chemistry analysis of the serum did not show any differences between WT and EN^{KO} mice.
AST: aspartate aminotransferase, ALT: alanine aminotransferase, AP: alkaline phosphatase, GGT: gamma-glutamyltransferase; CHE: cholinesterase; TG: triglycerides; Chol: cholesterol. Data are shown as mean ± s.e.m.

Supplementary Figure 5
Mogler et al.

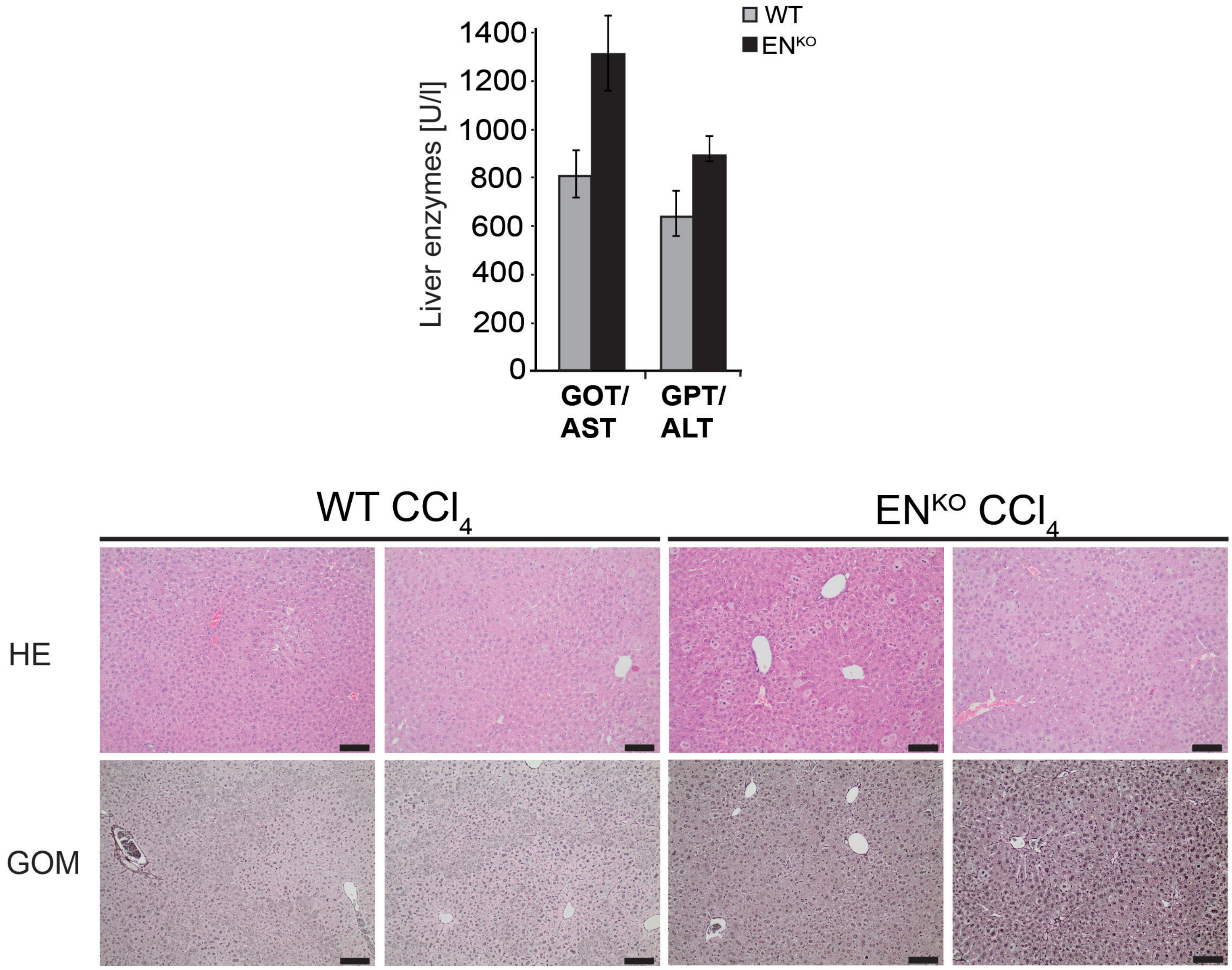

Liver serum tests (GOT/AST and GPT/ALT) and liver histology (HE, Gomoorhi silver stain) after single (high dose) i.p. injection of CCL_4.
Data shown as ± s.e.m.

Supplementary Figure 6
Mogler et al.

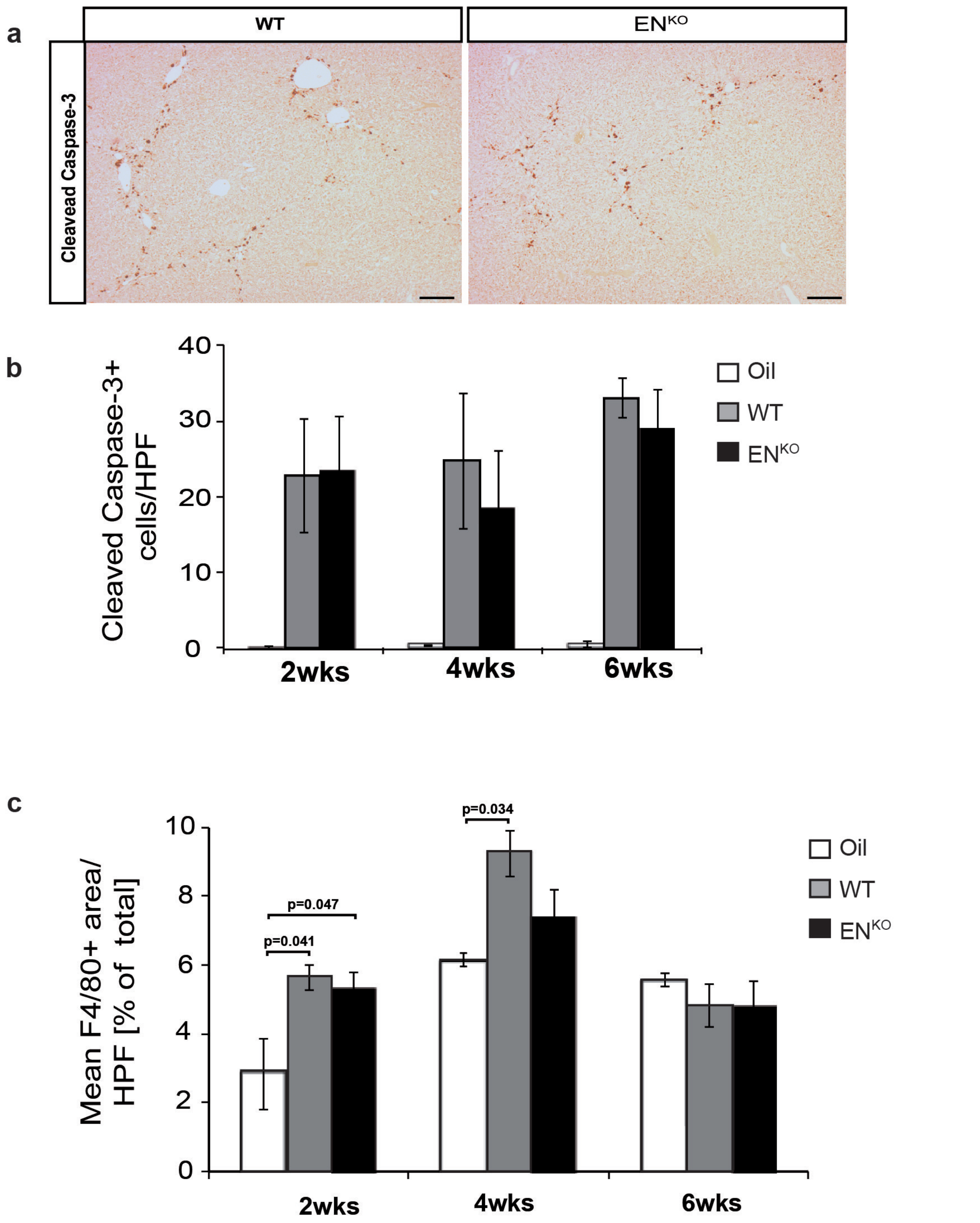

(a-b) Immunhistochemicals stainings and quantitation of cleaved caspase 3 in wildtype and EN^{KO} mice after 2,4 and 6 weeks of CCl_4 treatment.
(c) Quantitation of F4/80+ macrophages in wildtype and EN^{KO} mice after 2,4 and 6 weeks of CCl_4 treatment.
Data shown as ± s.e.m. p-value (significant <0.05) determined by student´s t-test.
Scale bar:100µm.

Supplementary Figure 7
Mogler et al.

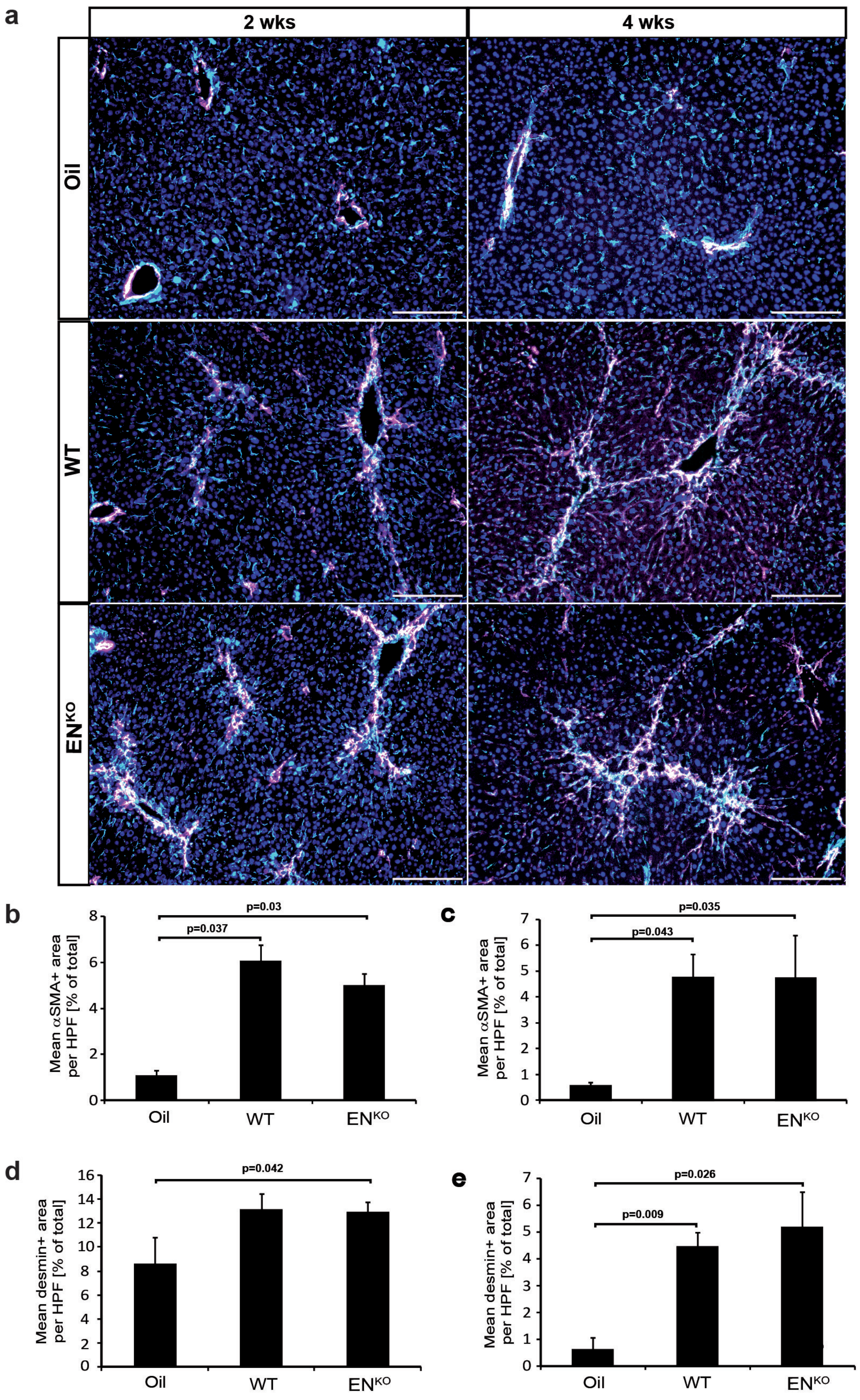

(a) Frozen liver sections of chronic oil- or CCl_4-treated WT and ENKO mice were double immunofluorescently stained for desmin (cyan) and αSMA (magenta) after 2 and 4 weeks. The αSMA+ (2 weeks: **b**, 4 weeks: **c**) and desmin+ (2 weeks: **d**, 4 weeks: **e**) areas were quantified per high power field (HPF) after chronic CCl_4 treatment. Scale bars: 200 µm; blue: Dapi; data are shown as mean ± s.e.m.; p-value (significant <0.05) determined by student´s t-test.

Supplementary Figure 8
Mogler et al.

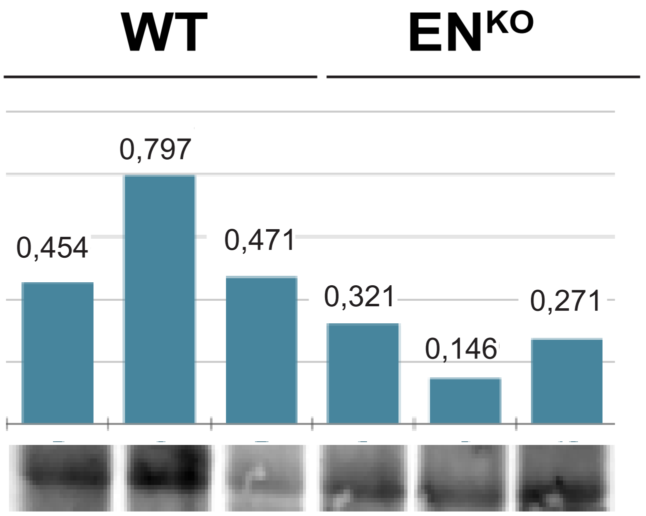

Quantitation of α SMA Protein in wildtype and EN^{KO} mice after 6 weeks of CCl_4 treatment (Normalized to total protein).

Supplementary Figure 9
Mogler et al.

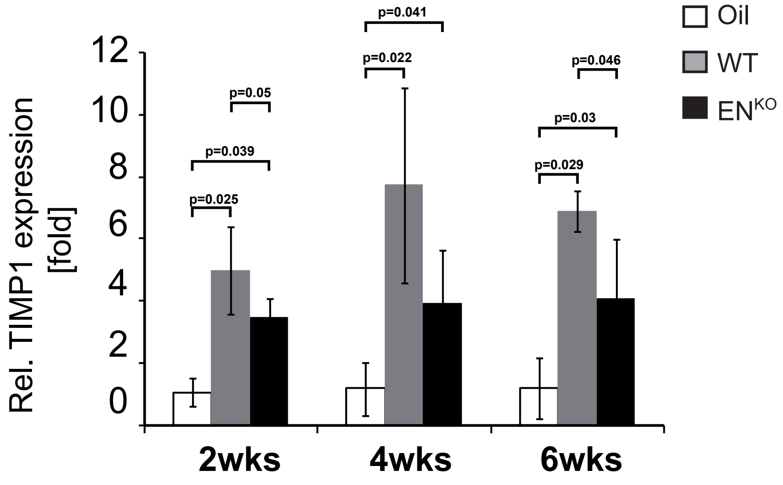

Q-RT-PCR of tissue inhibitor of metalloproteinase 1 (TIMP1) 2,4,6 weeks after CCl_4 treatment. Data shown as ± s.e.m.
p-value (significant <0.05) determined by student´s t-test.

Supplementary Figure 10
Mogler et al.

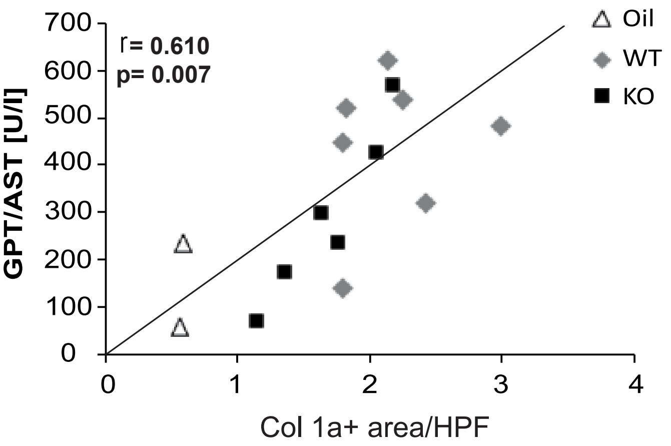

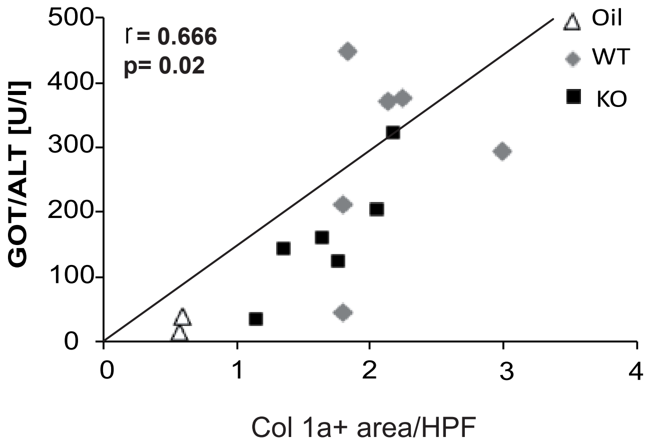

Positive correlation of serum liver enzymes GPT and GOT with regard to collagen 1a positive area/HPF
r = correlation coefficient. p-value (significant < 0.05) determined by Pearson´s bivariate correlation test.

Supplementary Figure 11
Mogler et al.

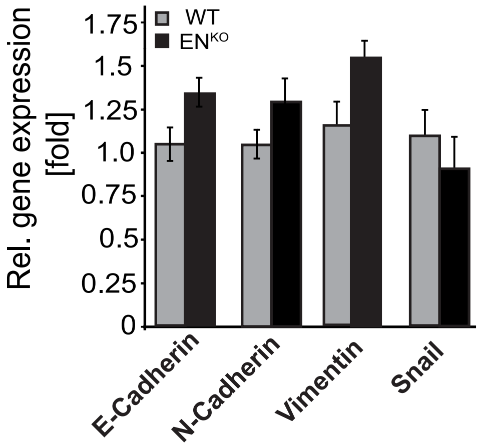

2 weeks CCl_4 treatment

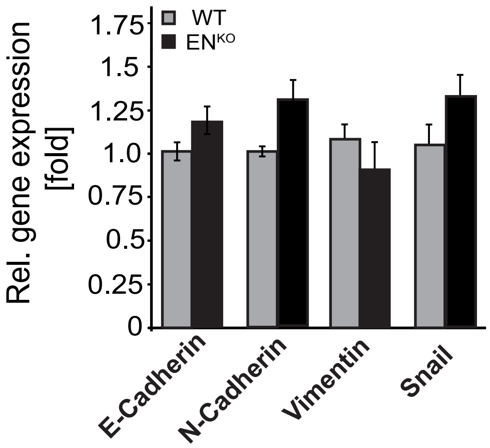

4 weeks CCl_4 treatemnt

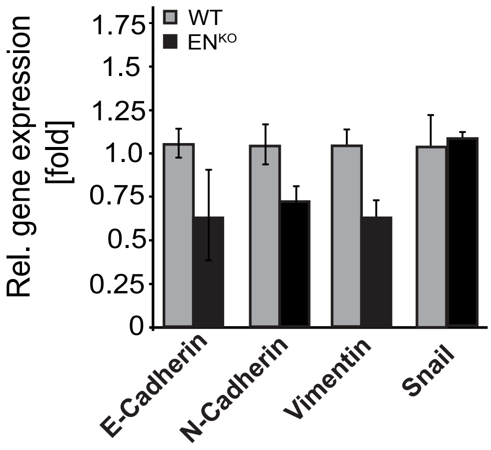

6 weeks CCl_4 treatment

Q-RT-PCR analysis of EMT markers of Endosialin wildtype and EN^{KO} mice after 2,4 and 6 weeks of CCl_4 treatment. Data shown as ± s.e.m.

Supplementary Figure 12
Mogler et al.

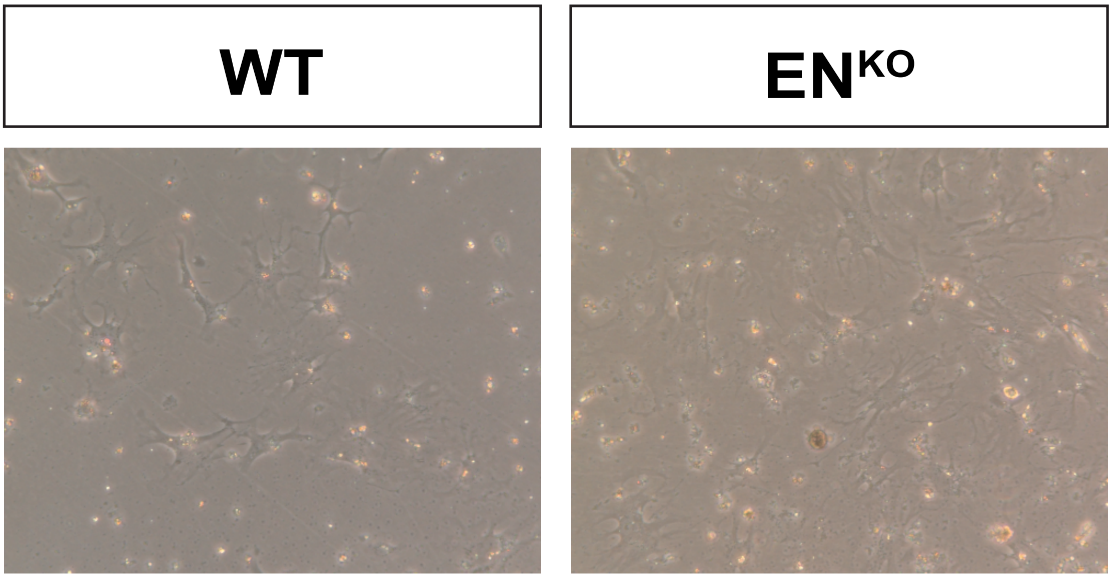

Cultivation of freshly isolated hepatc stellate cells from either wildtype or Endosialin knock-out mice over a period of eight days did not show significant morphological differences. Magnification: 10x (bright field).

Supplementary Figure 13
Mogler et al.

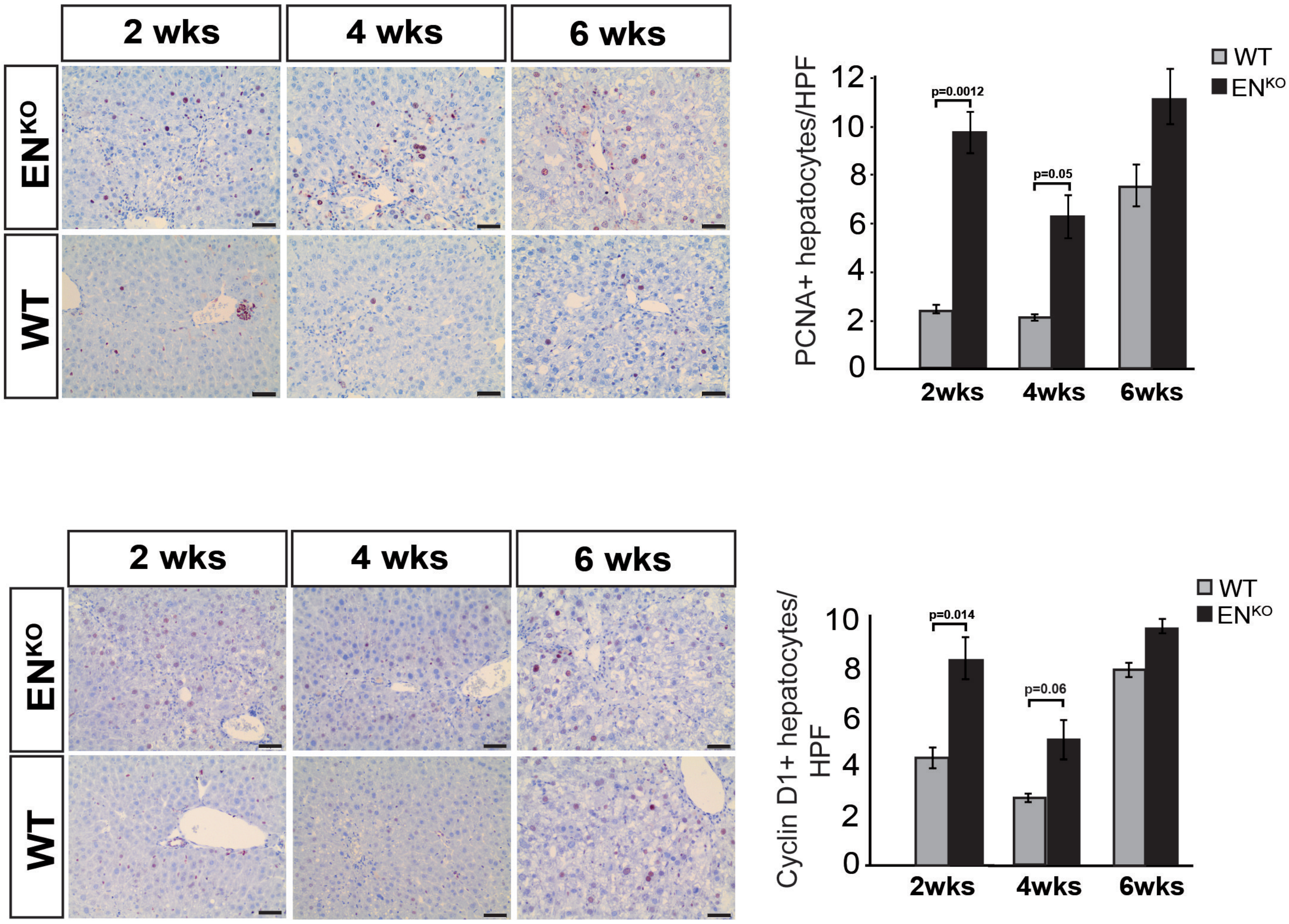

Immunohistochemical stainings of murine livers after 2, 4 and 6 weeks of CCl_4 treatment against proliferating cellular nuclear antigen (PCNA) and Cyclin D1.
Data are shown as mean ± s.e.m.; pvalue (significant <0.05) determined by student´s t-test.
Scale bars: 50 µm;

Supplementary Figure 14
Mogler et al.

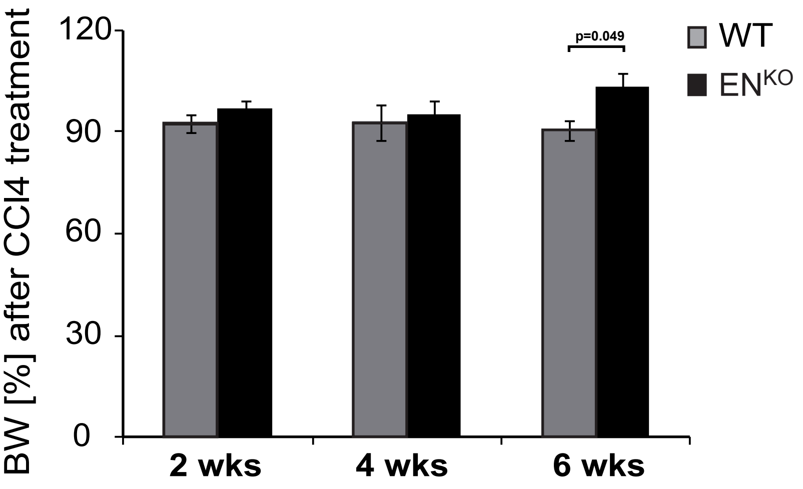

WT and EN^{KO} mice were treated with CCl_4 for 2, 4, and 6 weeks. The ratio of the initial body weight to final body weight after treatment is shown. Data are shown as mean ± s.e.m; p-value (significant <0.05) determined by student´s t-test.
BW= body weight. Wks=weeks.

Supplementary Figure 15
Mogler et al.

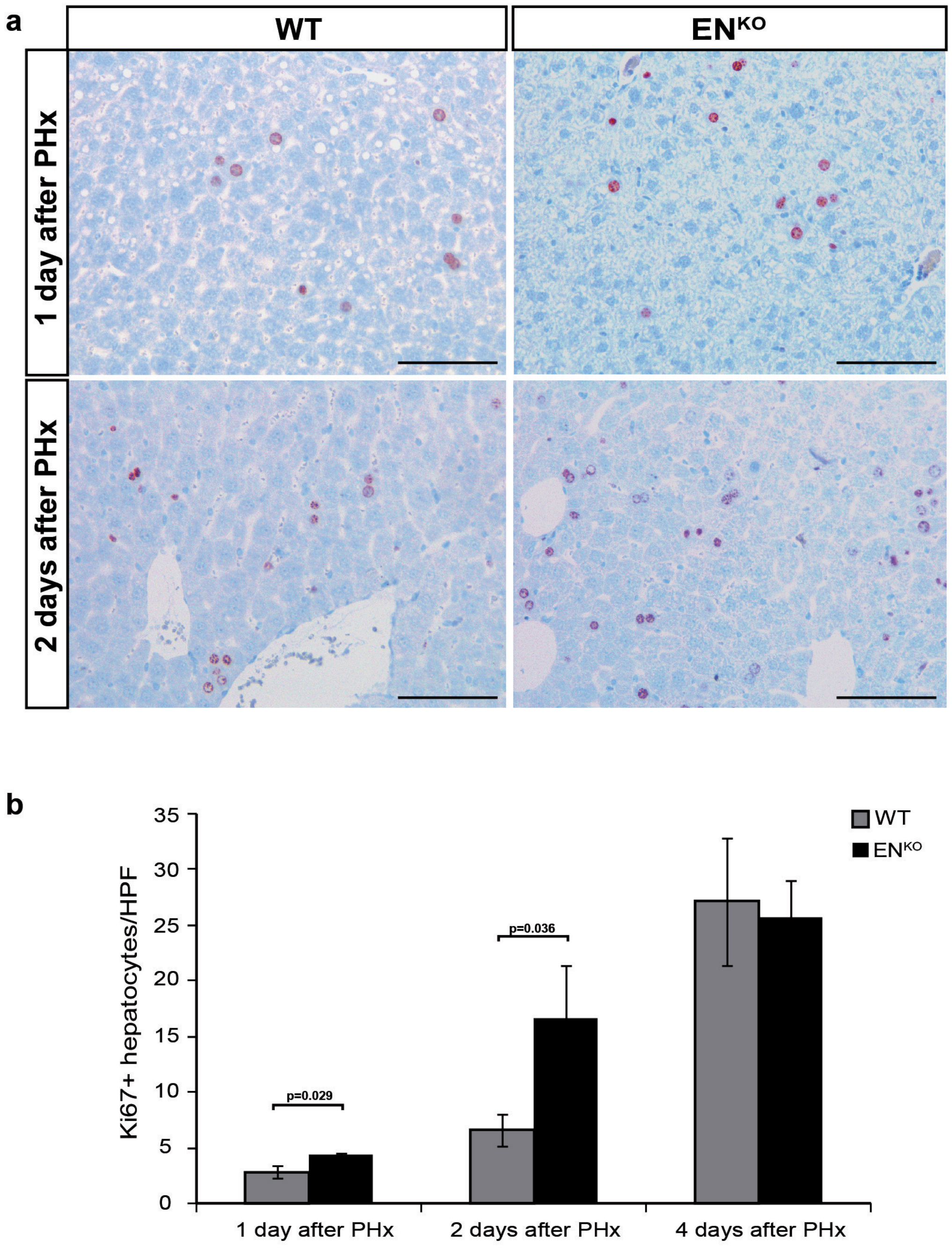

One third partial hepatectomy experiments were performed in WT and EN^{KO} mice. Proliferation was assessed by Ki67 immunohistochemistry on formalin-fixed paraffin embedded liver sections. **(a)** Representative pictures of WT mice and EN^{KO} mice at 1 and 2 days after operation **(b)** Ki67 quantifications for 1,2 and 4 days are shown. Scale bars: 50 µm; data are shown as mean ± s.e.m.; p-value (significant <0.05) determined by student´s t-test.

Supplementary Figure 16
Mogler et al.

a

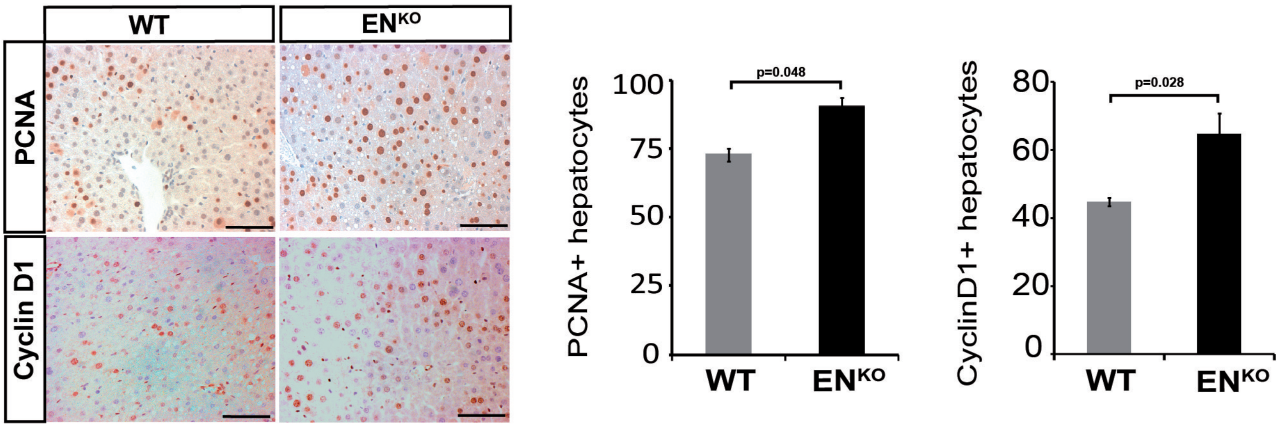

b

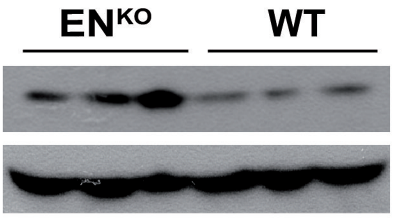

(a) Immunohistochemical stainings of murine livers 2 days after partial hepatectomy against proliferating cellular nuclear antigen (PCNA) and Cyclin D1. Scale bar: 100µm
Data are shown as mean ± s.e.m.; p-value (significant <0.05) determined by student´s t-test
(b) Total PCNA protein of wildtype and EN^{KO} livers 2 days after PHx.

Supplementary Figure 17
Mogler et al.

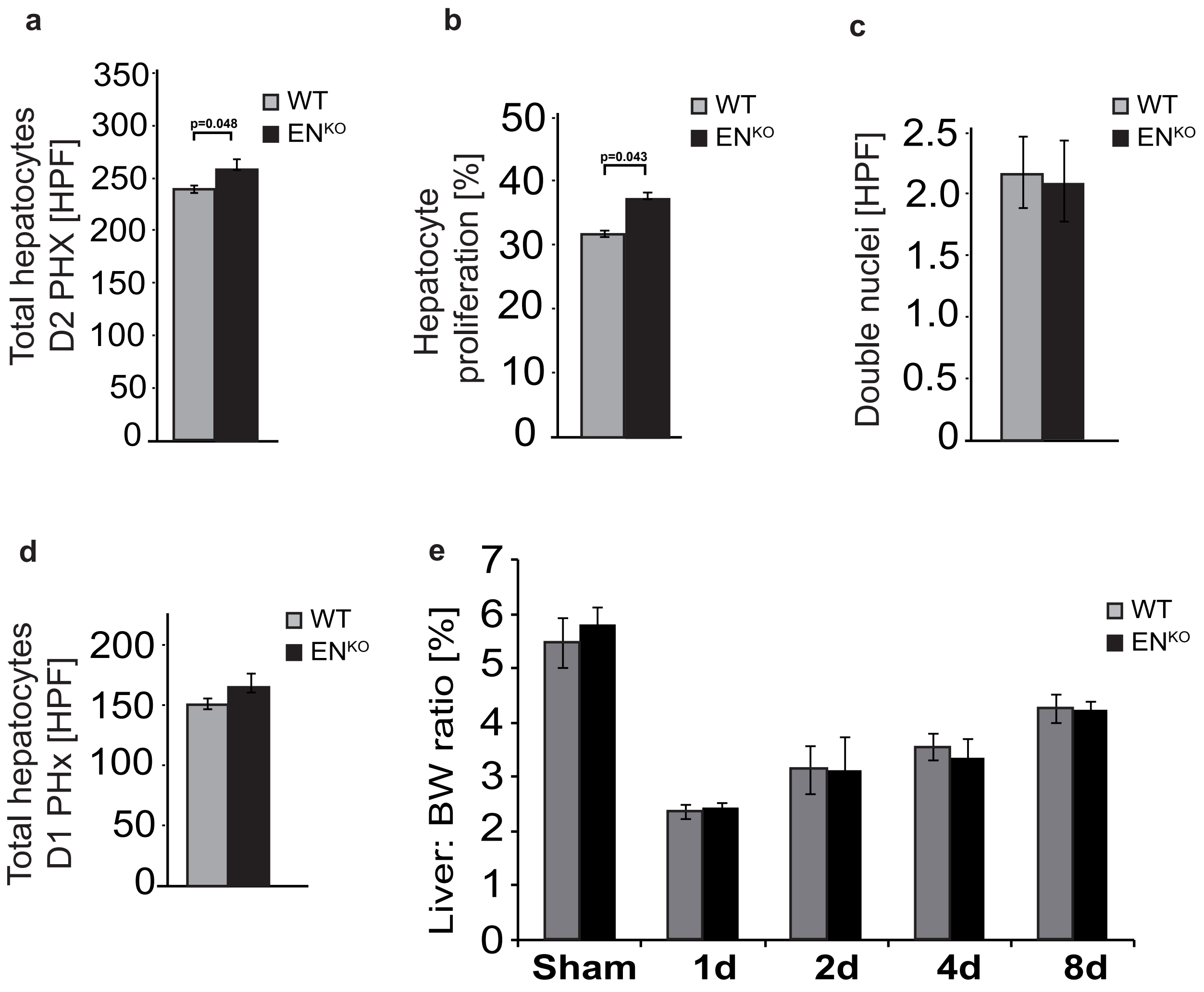

(a-c) Total hepatocytes/high power field, over all hepatocyte proliferation and number of double nuclei/high power field from Endosialin knockout and wildtype hepatectomized mice (all 2 days after PHx). **(d)** Total hepatocytes/HPF one day after PHx **(e)** Liver to body weight ratio of Endosialin wiltype and knockout hepatectomized and sham operated mice. Data are shown as mean ± s.e.m.; p-value (significant <0.05) determined by student´s t-test.

Supplementary Figure 18
Mogler et al.

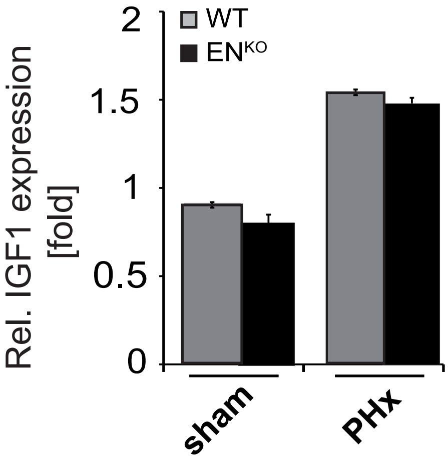

Q-RT-PCR analysis of IGF1 in sham operated wildtype and Endosialin knockout mice and 2 days after PHx. Data are shown as mean ± s.e.m.

Supplementary Figure 19
Mogler et al.

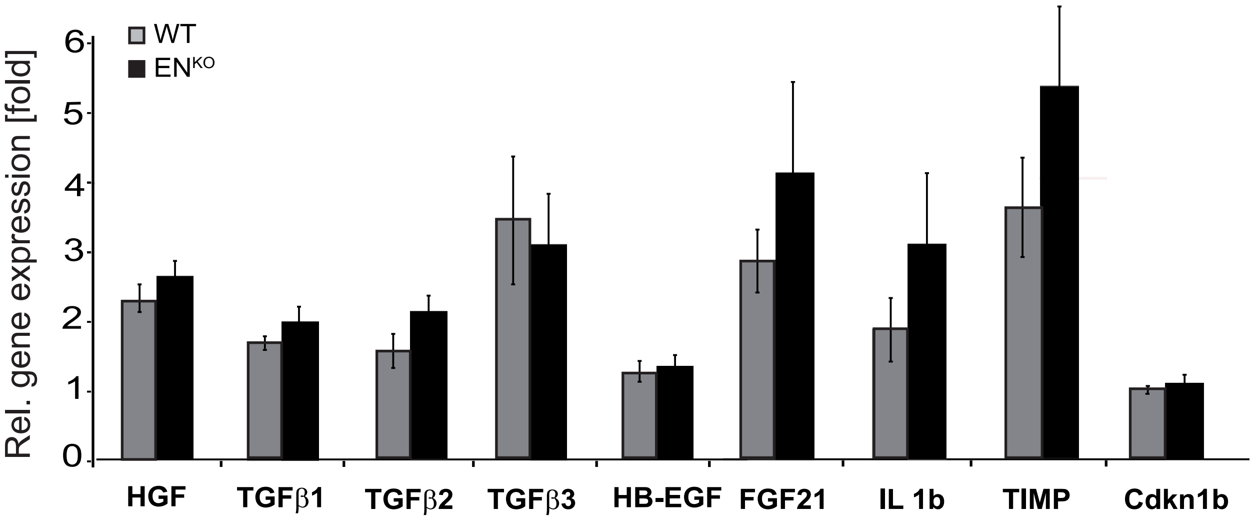

Q-RT-PCR analysis of hepatocyte mitogens hepatocyte growth factor (HGF), transforming growth factor (TGF), heparin-binding egf-like growth factor (HB-EGF), fibroblast growth factor (FGF 21), Interleukin 1b, Tissue inhibitor of metalloproteinase (TIMP) and cyclin dependent kinase 1b (Cdkn1b). Data shown as ± s.e.m.

Supplementary Figure 20
Mogler et al.

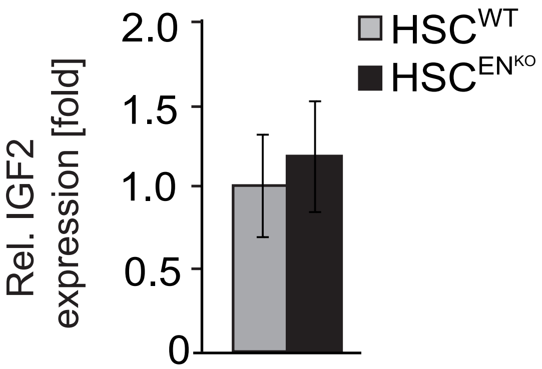

Q-RT-PCR analaysis of IGF2 in HSC from WT and EN^{KO} mice 8 days after cultivation. Data are shown as mean ± s.e.m.

Supplementary Figure 21
Mogler et al.

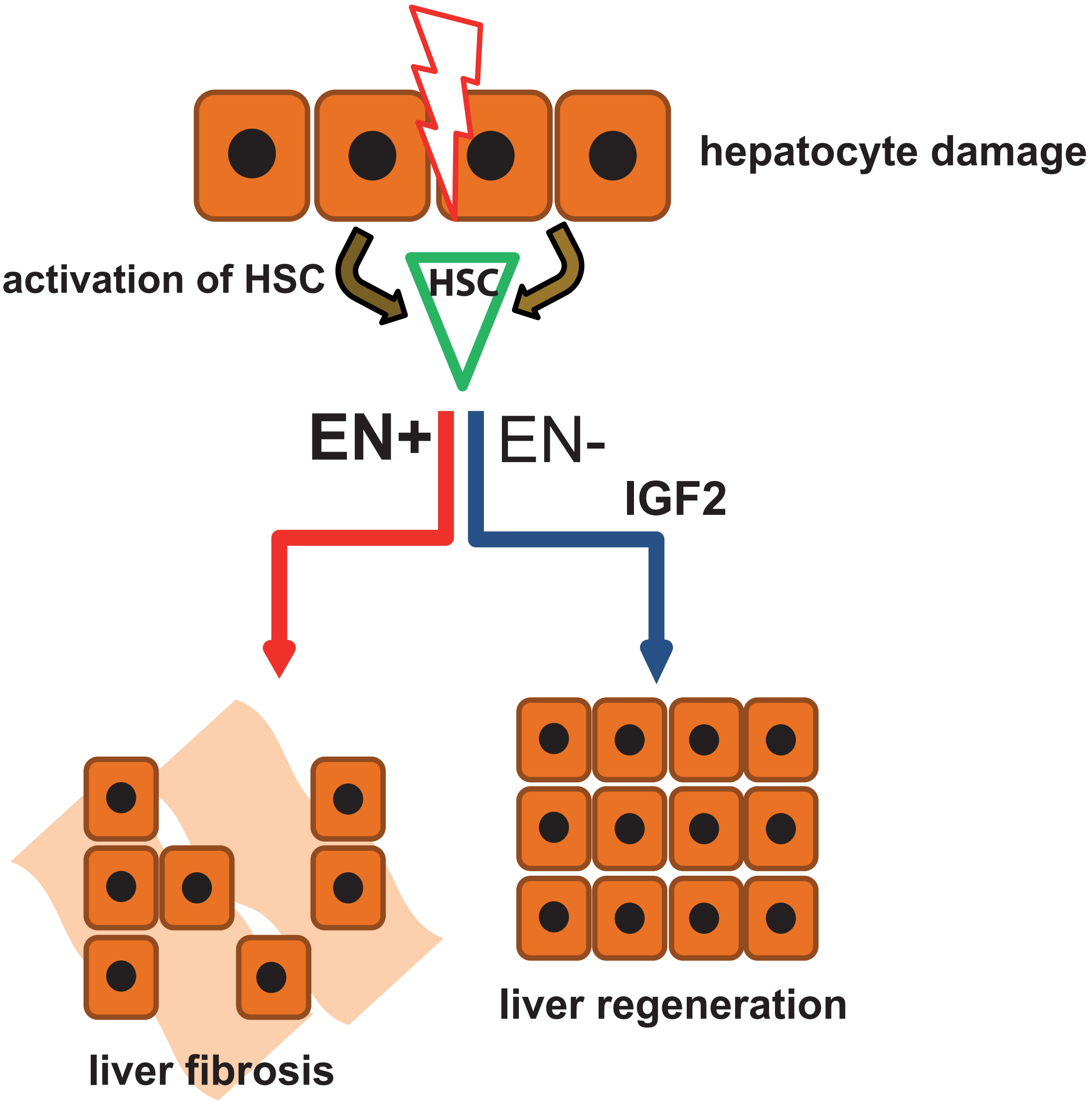

Conceptional model of Endosialin functionality in liver health and disease. Upon liver damage hepatic stellate cell (HSC) activation leads to fibrosis in the presence of Endosialin (EN+). In the absence of Endosialin (EN-), IGF2 mediated liver regeneration predominate the wound healing response.

Online supplement - Methods

Cell isolations

Hepatocytes were isolated from healthy C57/Bl6 mice at an age of 10-15 weeks. After perfusion with EGTA buffer-1, the liver was subsequently perfused with 30 ml of collagenase buffer-1 at same pump velocity. During perfusion, the liver was moisturized with suspension buffer or isotonic 0.9% NaCl. The liver capsule was opened with forceps to release the cells, and the obtained cell suspension was filtered through a 100 µm cell strainer. Suspension buffer was used to adjust to a final volume of 40 ml. Cells were centrifuged at 50 g for 2 min at room temperature, and the supernatant was discarded. Cell pellet was resuspended a final volume of 40 ml. Cells were again centrifuged and suspension buffer was stepwise added to a final volume of 10 ml.

Liver sinusoidal endothelial cells (LSEC) and Kupffer cells (KC) were isolated from C57/Bl6 mice at an age of 10-15 weeks. Liver perfusion was performed using Liver Digestion Medium (Invitrogen). The supernatant was filled up with PBS and centrifuged at 300 g for 10 min at 4°C. After washing, the cell pellet was resuspended in 2 ml RPMI, and 8 ml of 30% Histodenz/PBS solution was added. The gradient was prepared by underlaying 2 ml RPMI with the cell suspension in a 15 ml tube and centrifuges at 1,500 g for 20 min at 4°C without brake. Following centrifugation, the white layer of low density cells at the interface was harvested and transferred to a new tube. Cells were washed twice with LSEC-buffer and centrifuged at 300 g for 10 min at 4°C. The cell pellet was resuspended in 90 µl LSEC-buffer, and cells were incubated for 15 min at 4°C with 10 µl of CD146 microbeads for LSEC isolations or CD11b microbeads for Kupffer cell isolations (Miltenyi). 2 ml LSEC-buffer was added and cells were centrifuged at 300 g for 10 min at 4°C. The supernatant was completely aspirated, and the remaining pellet was resuspended in 500 µl of LSEC-buffer. For magnetic-activated cell sorting (MACS), respective columns were placed to a MACS separator, and columns were rinsed with 500 µl LSEC-buffer. Cell suspension was applied to the columns and washed three times with 500 µl LSEC-buffer. The column was finally removed from the separator, placed on a new collection tube, and bound cells were eluted after addition of 1 ml LSEC-buffer by firmly pushing the plunger into the column.

HSC were isolated from healthy, oil, and CCl_4 treated C57/Bl6 male mice at an age of about 20 weeks. Using preheated SC1, SC2+ PronaseE, SC2+ CollagenaseD, and SolutionD buffers. Mice were anaesthetized by intraperitoneal injections of a mixture of Ketamine (100 mg/kg) and Xylazine (10 mg/kg) diluted in isotonic 0.9% NaCl and the inferior vena cava (IVC) was exposed. A 25G (0.5 mm x 12.7 mm x 305 mm) butterfly needle was inserted into the IVC and fixed using a vascular clamp. Perfusion was subsequently carried out by SC1 for 2.5 min at a pump velocity of 10 ml/min, followed by SC2+PronaseE for 3.5 min at a pump velocity of 5 ml/min, and finally SC2+CollagenaseD for 7 min (5 ml/min). The perfused liver was harvested and stored in DMEM, 4.5 g glucose on ice. Livers from further 2-3 mice were harvested after perfusion. All livers were completely minced in pre-warmed solution (40°C), and pH was measured (7.35-7.4). The cell suspension was transferred into a breaker and stirred at 100-300 rpm for about 5 min at 37°C. Afterwards, the suspension was filtered through a sterile nylon mesh (120 µm), subsequently transferred into four 50 ml tubes with DMEM, 4.5 g glucose, and centrifuged at 50 g for 3 min at 4°C. The supernatants were transferred into new 50 ml tubes and washed again with DMEM, 4.5 g glucose. Following

centrifugation at 2000 g for 10 min at 4°C, the supernatants were discarded, pellets were resuspended in 10 ml DMEM, 4.5 g glucose +150 µl DNase I (2 mg/ml), combined in one 50 ml tube, and filled up with DMEM, 4.5 g glucose. The cell suspension was centrifuged at 2000 g for 10 min at 4°C. The resulting pellet was resuspended in an appropriate volume and mixed with 30% Nycodenz solution to prepare a 15.6% Nycodenz cell suspension. GBSS/B and 30% Nycodenz solution was mixed to yield a 13.2% solution, a 11.5% solution, and a 8.26% solution, respectively. The density gradient was subsequently prepared by overlaying the 15.6% Nycodenz-cell solution with the 13.2% solution, followed by the 11.5% solution, the 8.26% solution, and finally ice-cold GBSS/B. Centrifugation was carried out at 1,400 g for 23 min at 4°C without brake. After centrifugation, the three layers 8.26%, 11.5%, and 13.2% below the GBSS/B were harvested and washed with DMEM, 4.5 g glucose (centrifugation at 2000 g, 10 min, 4°C). Supernatant was discarded and cell pellet was resuspended in DMEM, 4.5 g glucose. Cells were counted using trypan blue. After isolations, cells were either seeded directly in 12-well µ-slides, or further purified by dynabead cell sorting. Therefore, the isolated cells were blocked in PBS/5% FCS buffer supplemented with murine IgG (1:20) for 30 min at 4°C (100 µl/1x10^7 cells). Cells were washed with 1 ml PBS/5% FCS by centrifugation at 900 g for 3 min at 4°C. Cell pellet was subsequently resuspended in PBS/5% FCS (100 µl/1x10^7 cells) supplemented with antibodies against CD45-PE (1:200), CD11b-PE (1:400), CD34-Pacific blue (1:100), CD31-FITC (1:500), and F4/80-PE (1:100). The antibody binding reaction was carried out for 45 min at 4°C in the dark, followed by washing with 1 ml PBS/5% FCS (900 g, 3 min, 4°C). Afterwards, the pellet was resuspended in PBS/5% FCS ($<10^7$ cells: 500 µl, 10^7-10^8 cells: 750 µl), mixed with vigorously vortexed dynabeads ($<10^7$ cells: 500 µl, 10^7-10^8 cells: 750 µl), and incubated on a rotator for 30 min at 4°C. Negative selection was performed by placing reaction tubes to a magnetic field. The clear supernatant was transferred to a new tube and washed with 500 µl PBS/5% FCS (900 g, 3 min, 4°C). Cell pellet was resuspended in 600 µl PBS/5% FCS. A 100 µl aliquot was seeded in 12-well µ-slides (Ibidi) in DMEM, 4.5 g glucose, 30% FCS, 1% P/S. The remaining 500 µl were centrifuged (900 g, 3 min, 4°C) and lysed by RLT lysis buffer supplemented with β-mercaptoethanol.

Cell culture of LX2 human HSC

The human HSC cells LX2 were originally generated by Scott Friedman´s lab. LX2 were cultured in 10 cm dishes with DMEM 1 g glucose, 2% fetal calf serum and 1% penicillin/streptomycin, at 37°C, 5% CO_2. Cells were passaged as necessary. Endosialinknockdown was achieved by GFP-labeled adenoviral mediated transfection of LX-2 cells using either non silencing or Endosialin-silencing virus. For selection of non-trasnfected cells, Puromycin was added to the cell culture medium.

Formalin-fixed paraffin-embedded/cryo tissues and sections

All organs and tissues were fixed in 4% PFA overnight. Samples were manually embedded in paraffin blocks and 2-4 µm thick tissue sections were prepared. HE stain was performed according to standard protocols and all slides were evaluated by at least one board certificated surgical pathologist. Tissues for cryo fixation were placed in cryomold immersed in OTC Cryo-medium, or alternatively on cork plates, and were snap frozen in liquid nitrogen or on dry ice. 7 µm sections were cut.

Immunohistochemistry

Paraffin sections were dewaxed and rehydrated through a graded ethanol series. Antigen unmasking was performed using a citrate buffer (0.01M sodium citrate pH 6) in a water bath at 95°C for 20 min followed by cooling in dest. H_2O. To block background activity of endogenous peroxidases, slides were treated with 3% H_2O_2 for 15 min. Slides were incubated 20 min at room temperature and further over night at 4°C with primary antibody in the appropriate buffer. Secondary antibody was incubated for 30-45 min at room temperature. Detection was performed via a biotin-peroxidase complex, in form of Vectastain ABC solution according to manufacturers' protocol. Sections were counterstained with Hematoxylin for 2 min, rinsed with running tap water for 10 min. A list of all primary and secondary antibodies used in this study can be found in table 1 and 2.

(Double) immunofluorescence stainings on cryosections

Cryosections were fixed for 10 min using methanol. Samples were treated with blocking solution for 1 h at room temperature. Primary antibodies were applied to sections in appropriate dilutions in blocking serum for 1 h at room temperature or 4°C over night. Secondary antibodies were added in appropriate dilutions in TBS-T for 1 h at room temperature. Nuclei were stained with the DNA intercalating agent Hoechst (Dapi) in a 1:2000 dilution for 10 min at room temperature. Samples were mounted with fluoromount. A list of all primary and secondary antibodies used in this study can be found in table 1 and 2.

Agarose gel electrophoresis

1% (w/v) agarose was dissolved 0,5x TAE buffer by heating. Before pouring the solution into a casting tray ethidium bromide 5 µl/100 ml was added. Samples were mixed with a 6x loading buffer, and the gel was run with 3-6 V/cm. DNA was visualized under UV-light.

RNA preparation from cells

RNA was purified from either isolated stellate cells or liver pieces by the Rneasy Mini Kit from Qiagen according manufacturer´s instructions. Concentration and purity was measured by analyzing 1 µl of sample with the RNA program of the Nanodrop. If necessary, samples were further diluted with Rnase-free water. A 260/280 ratio of ≥2 represented protein-free RNA. Purified RNA was stored at -80°C.

RNA preparation from organs

RNA was purified from either mouse or human livers pieces or 10-20 collected 10 µm cryo sections. Liver material was lysed with 1 ml TRIzol and completely homogenized. Homogenized samples were incubated for 5 min at room temperature. In order to separate proteins from RNA and DNA, 200 µl of chloroform was added in a peqGold PaseTrap A tube with homogenized material and chloroform were mixed by manually for 20 sec, incubated for 5 min at room temperature, and finally centrifuged at 12,000 g for 15 min at 4°C. The upper, aqueous phase was transferred to a new Rnase-free eppendorf tube. RNA was precipitated after adding 500 µl isopropanol, tube inverting, and incubation for 10 min at room temperature, followed by centrifugation at 12,000 g for 10 min at 4°C. Pelleted RNA was washed with 1 ml of 75% ethanol, vortexed, and centrifuged at 7,500 g for 5 min at 4°C. Pellet was air dried for 5 min at room temperature and resuspended with 200 µl of 55-60°C pre-warmed Rnase-free water. 8 µl of 5 M NaCl, and 200 µl phenol-chloroform-

isoamylalcohol (25:24:1) were added to the 200 µl RNA solution, and mixed by vortexing. After centrifugation at 12, 000 g for 15 min at 4°C, the upper, aqueous phase was transferred into a new Rnase-free eppendorf tube. RNA was once more precipitated with 200 µl isopropanol and further handeled as already described. Pelleted RNA was finally dissolved in 30-50 µl of 55-60°C pre-warmed Rnase-free water. Purified RNA was stored at -80°C.

RNA isolation from LSEC and KC

Cells were lysed with 1 ml TRIzol, homogenized by pipetting, and incubated for 5 min at room temperature. The homogenization was transferred to a peqGold PaseTrap A tube. 200 µl chloroform was added, the tube was shaken for 15 sec, incubated for 3 min at room temperature, and finally centrifuged at 12,000 g for 15 min at 4°C. The upper, aqueous phase was transferred to a new Rnase-free tube and mixed with 1.5 volumes of 100% ethanol. RNA purification was performed with the miRNeasy mini kit from Qiagen according manufacturer´s instructions. Purified RNA was stored at -80°C.

cDNA generation

cDNA generation was done by using the Quantitect® Reverse Transcription Kit from Qiagen according manufacturer’s instructions. cDNA was stored at -20°C for further use.

Quantitative real time-PCR (qRT-PCR)

Relative gene expression analysis was performed using qRT-PCR. The Taqman mono-color hydrolysis probe method (Applied Biosystems) was used to detect differences in the amount of mRNA transcript levels. TaqMan probe sets were purchased from Applied Biosystems.

Preparation of protein lysates from organs

Protein lysates from organs were prepared from frozen organs or collected material from cryo sectioning. Liver material was lysed by smashing in 0.5-1 ml NP40 lysis buffer with phosphatase inhibitor orthovanadate (concentration 2mM). To sediment cellular debris, cell suspension was centrifuged for 10 min at 12,000 rpm, 4°C and cleared lysate stored at -20°C. Protein concentrations determined by using Bradford- or the BCA-assay.

Western blot analysis

Proteins were separated electrophoretically according to their molecular weight in 10% SDS-polyacrylamide gels. 10 µl of protein standard was loaded. Electrophoresis was carried out at constant 80 V until samples reached the separating gel, then voltage was increased to 120 V until the end of gel separation. Transfer of proteins from gel to membrane was carried out at constant 400 mA in a cooled chamber for 70 min. After transfer, membranes were washed once in TBST, and then blocked for 1 h with 3% BSA (w/v) in TBS-T. Membranes were incubated with the primary antibody with gentle rocking overnight at 4°C. Incubation with appropriate secondary antibody conjugated with horseradish peroxidase in TBS-T, 1% BSA (w/v) for 1 h at RT. For chemiluminescent detection of protein bands, ECL solution was used according to manufacturers’ protocol. Blotting for β-actin was used as loading control.

Table 1: Primary antibodies

target protein	species	application	company
Actin	goat	1:2,000 WB	Santa Cruz
αSMA-Cy3	mouse	1:200 staining	Sigma-Aldrich
αSMA	mouse	1:250 staining	DAKO
CD11b-PE	rat	dynabead	eBioscience
CD34-pacific blue	rat	dynabead	eBioscience
CD45-PE	rat	dynabead	BD Pharmingen
Cleaved caspase 3	rabbit	1:100 staining	Cell signaling
Collagen-1a	rabbit	1:1,000 staining	AbD Serotec
CyclinD1	rabbit	1:25 staining	Cell signaling
Desmin	rabbit	1:100 staining	Abcam
Caldesmon	mouse	1:50 staining	Dako
Endosialin (FB5)	mouse	10 µg/ml staining, WB	FB5-hybridoma cells
Ki-67	rat	1:100 staining	Dako
PCNA	rabbit	1:250 staining, WB	Abcam
PDGFRβ	rabbit	1:1,000 WB	Cell signaling

Table 2: Secondary antibodies

target protein	species	application	Company
anti-goat HRP	rabbit	1:5,000 WB	DAKO
anti-mouse-HRP	rabbit	1:5,000 WB	DAKO
anti-mouse-HRP	goat	1:250 staining	Jackson/Dianova
anti-rabbit HRP	goat	1:5,000 staining, WB	DAKO
anti-rabbit A488	goat	1:250 staining	Invitrogen
anti-rabbit A488	donkey	1:250 staining	Invitrogen
anti-rabbit biotinylated	goat	1:250 staining	DAKO
anti-rat biotinylated	goat	1:250 staining	Vector labs
anti-rabbit-AP	donkey	1:250 staining	Jackson/Dianova
anti-rat A488	goat	1:250 staining	Invitrogen
Streptavidin A546	goat	1:500 staining	Invitrogen
anti-mouse A488	goat	1:500 staining	Invitrogen
