# Supplementary material for: Hepatic stellate cell-expressed endosialin balances fibrogenesis and hepatocyte proliferation during liver damage
Source: EMBO Mol Med. 2015 Feb 13;7(3):332–8. doi: 10.15252/emmm.201404246 (PMC4364949; doi:10.15252/emmm.201404246)
Supplement: Supplementary file 2 [file emmm0007-0332-sd2.pdf]

Hepatic stellate cell expressed Endosialin balances fibrogenesis and hepatocyte proliferation during liver damage

Carolin Mogler, Matthias Wieland, Courtney König, Junhao Hu, Anja Runge, Claudia Korn, Eva Besemfelder, Katja Breitkopf-Heinlein, Dorde Komljenovic, Steven Dooley, Peter Schirmacher, Thomas Longerich, Hellmut G. Augustin

Corresponding author: Hellmut Augustin, Medical Faculty Mannheim, University of Heidelberg, and German Cancer Research Center Heidelberg

Review timeline:

Submission date:	09 May 2014
Editorial Decision:	17 June 2014
Revision received:	17 November 2014
Editorial Decision:	12 December 2014
Revision received	07 January 2015
Accepted:	13 January 2015

Transaction Report:

(Note: With the exception of the correction of typographical or spelling errors that could be a source of ambiguity, letters and reports are not edited. The original formatting of letters and referee reports may not be reflected in this compilation.)

Editor: Roberto Buccione

1st Editorial Decision 17 June 2014

Thank you for the submission of your manuscript to EMBO Molecular Medicine. We are sorry that it has taken longer than usual to get back to you on your manuscript.

In this case we experienced unusual difficulties in securing three appropriate reviewers and then obtaining their evaluations in a timely manner. Further to this, I wished to discuss with my colleagues and the Chief Editor, who was not immediately available.

As you will see, the Reviewers find merits in your manuscript but raise significant issues. I will not dwell into much detail, as their comments are thorough. I would like, however, to highlight a few main points.

Firstly, all three Reviewers clearly agree that your manuscript suffers from a lack of sufficient mechanistic insight in general and specifically into the mode of action of endosialin (EN). This is clearly a major and shared item of dissatisfaction that I'm afraid we agree upon. There are also

© EMBO

specific additional concerns from each Reviewer.

Reviewer 1 contends that the effects of EN knockout are modest and suggests that additional approaches should be employed to confirm the conclusions including blinded pathology grading, liver function tests, etc.

Reviewer 2 notes that your conclusions on the role of EN are based on a whole body knockout model and that a conditional knockout would be required to draw firm conclusions regarding the specific involvement of HCSs.

Reviewer 3 is especially concerned that the molecular link between EN knockout and IGF2 is not demonstrated. This Reviewer also notes that it would be of great interest to test EN targeting drugs in vivo. Finally, s/he mentions that some conclusions are not sufficiently supported by the data.

In conclusion, while publication of the paper cannot be considered at this stage, given the potential interest of your findings and the fact that the Reviewers, although highly critical, did not call for outright rejection at this time, we have decided to give you the opportunity to address the above concerns.

We are thus prepared to consider a substantially revised submission, with the understanding that the Reviewers' concerns must be addressed with additional experimental data where appropriate and that acceptance of the manuscript will entail a second round of review. While Reviewer 2's point that a conditional knockout would be better is well taken, we appreciate that to carry out such experimentation would be perhaps further reaching. Nevertheless, in addition to addressing the other concerns, I would encourage you to tackle the specificity of action issue raised by Reviewer 2 as far as realistically possible. In addition, if you could show, as Reviewer 3 suggests, the efficacy of EN-targeted drugs in vivo, this would increase the translational impact of your work while decreasing the level of mechanistic insight required.

I understand that if you do not have the required data available at least in part, to address the above might entail a significant amount of time, additional work and experimentation and might be technically challenging, I would therefore understand if you chose to rather seek publication elsewhere at this stage. Should you do so, we would welcome a message to this effect.

Please note that it is EMBO Molecular Medicine policy to allow a single round of revision only and that, therefore, acceptance or rejection of the manuscript will depend on the completeness of your responses included in the next, final version of the manuscript.

As you know, EMBO Molecular Medicine has a "scooping protection" policy, whereby similar findings that are published by others during review or revision are not a criterion for rejection. However, I do ask you to get in touch with us after three months if you have not completed your revision, to update us on the status. Please also contact us as soon as possible if similar work is published elsewhere.

I look forward to seeing a revised form of your manuscript as soon as possible.

***** Reviewer's comments *****

Referee #1 (Remarks):

This is a fascinating and novel study that identifies endosialin (EN) to be induced in hepatic stellate cells during liver injury and to mediate paracrine inhibition of hepatocyte regeneration in the injured

liver. A candidate approach suggested that IGF2 is an EN-regulated factor that may be responsible for the observed negative impact of EN on regeneration, although this idea was not formally tested.

Overall the work addresses an important emerging theme of the interplay between regenerative and fibrogenic mechanisms in the injured liver and supports the concept that stimulating hepatocyte regeneration may prevent fibrosis. Moreover, the study supports recent published evidence that activated liver myofibroblasts may actively repress regeneration, although it is important to note that these cells have also been proposed to be stimulators of hepatocyte proliferation.

The strengths of the study are:

1. The use of well-established liver injury and regeneration models to demonstrate up regulation of EN in the injured liver and combined use of EN knockout mice to show that the lectin plays a pro-fibrogenic and anti-regenerative role.

2. The application of histological, immunohistochemical and biochemical measurements to demonstrate effects of EN knockout on liver physiology and pathology.

The main weaknesses of the study that need to be addressed by the authors are:

1. The effects of EN knockout on fibrosis appear modest and many of the measurements are either at the mRNA level or from morphometric analysis of stained liver sections. Ideally changes in the expression of key proteins such as aSMA should be shown by Western blotting. Given the modest effect of EN knockout on fibrosis it would be preferred that the authors carry out a blinded pathology grading of liver damage and fibrosis using a well-accepted grading systems (e.g. Metavir).

2. The authors should examine the effects of EN knockout on serum liver function tests (ALT, AST, ALP). If as they suggest EN knockout is hepatoprotective then at least in the chronic CCl4 model this should be reflected in improved LFTs. It would also be important to look at LFTs in the acute CCl4 model at an early time point (6-12hrs) in order to rule out a direct effect of EN on the CCL4-induced liver damage mechanism.

3. The weakest part of the study is its mechanistic component, i.e. how is EN expressed on hepatic myofibroblasts modulating hepatocyte regeneration. The authors identify IGF2 as a potential mediator but do not formally test this idea either in vivo or in vitro. Without formally testing if IGF2 mediates the effects of EN this important component of the study is superficial at best. It is also difficult without this mechanistic information to envisage how and why EN expressed on myofibroblasts should regulate hepatocyte proliferation. What is the cellular source of the IGF2 and how is regulated by EN are further important questions. Furthermore the candidate approach is limited, ideally the authors should have presented genome-wide data which is now cheap enough and available to all labs.

Referee #2 (Comments on Novelty/Model System):

The evidence is largely based on data derived in a global knockout of endoelasin, making it difficult to know how much of the phenotypic alterations are directly due to loss of endoelasin in stellate cells themselves. A stellate cell-specific knock out would be required to do this.

Assuming loss of stellate cell-derived endoelasin is driving the phenotype, more work is needed to delineate the mechanisms. For example, in what way does loss of endoelasin change stellate cells? Does stellate cell conditioned medium from wild type and knock out stellate cells differentially impact hepatocyte proliferative activity?

Referee #2 (Remarks):

The studies document hepatic stellate cell (HSC) expression of endoelasin and show that global knockouts of endoelasin are somewhat protected from carbon tetrachloride-induced liver fibrosis and demonstrated enhanced liver regeneration after partial hepatectomy (PH). However, the mechanisms for these outcomes remain uncertain. Key issues follow:

1 - The evidence for decreased liver fibrosis and increased liver regeneration is based on data derived in a global knockout of endoelasin, making it difficult to know how much of the phenotypic alterations are directly due to loss of endoelasin in stellate cells themselves. A stellate cell-specific knock out would be required to do this.

2 - Assuming loss of stellate cell-derived endoelasin is driving the phenotype, more work is needed to delineate the mechanisms. For example:

a) In what way does loss of endoelasin change stellate cells?

b) Does stellate cell conditioned medium from wild type and knock out stellate cells differentially impact hepatocyte proliferative activity?

c) Does endoelasin (or its deficiency) influence epithelial-to mesenchymal or mesenchymal to epithelial transitions?

Referee #3 (Comments on Novelty/Model System):

Please see my detailed critics

Referee #3 (Remarks):

In their work, the authors addressed the possible function of Endosialin, a c-type lectin, in models of liver disease. The authors provide evidence that Endosialin, which seems to be expressed mainly by hepatic stellate cells, promotes liver fibrosis but in turn suppresses proliferation of hepatocytes, making it an attractive candidate for pharmacological targeting in chronic liver disease.

From a technical point of view, these are clean and solid data. My main concern however is that the whole manuscript appears quite preliminary, lacking a conclusive molecular explanation for the potentially very interesting effect of Endosialin-knockout in these models. These are my specific concerns:

1. Most importantly, there is no explanation at present how Endosialin (EN) on HSC controls liver fibrosis. I do not see any experiment providing data for this functional question.

2. The molecular linkage between EN knockout and the supposed target gene IGF-2 is not convincing at all and at this stage precludes publication in EMM. This assumption is solely based on one single RT-PCR. From our experience, IGF-2 is rather a marker of proliferation in liver models (similar to H-19) and is upregulated in proliferating liver cells in most models we looked at. The hypothesis that EN in HSC directly controls IGF-2 (how?) which again is the driving factor for hyper proliferation (how) is not backed up (and to my point of view rather unlikely). A whole new

set of experiments is needed from my point of view to provide a functional explanation for these important questions

3. Given the clinical focus of EMM, it would be interesting if the authors tested one of the available drugs targeting EN in their mouse model of liver fibrosis.

4. Some interpretations in the manuscript are not sufficiently backed up by the data (see below)

Minor critics:

1. The results part starts with a quite confusing section. Clearly distinguish introduction and results.

2. I understood that HSC were separated by density gradient centrifugation. This method is inferior to more advanced techniques, e.g. combinations with Facs-based cell sorting in terms of purity. It might make sense to check expression data in pure primary HSC.

3. The Immunostainings for SMA and EN are not completely clear to me. Are all SMA+ cells EM+, e.g. in cirrhosis? Are also other cells positive? Provide double stainings.

4. The statement on equal liver damage in both experimental groups is based solely on cleavage of Casp-3. This is clearly not sufficient, since other forms of cell death might be much more relevant in CCl_4 than apoptosis. Provide serum AST, ALT, GLDH data and necrosis analysis.

5. Are all shown figures representative for the statistics? This appears critical in Fig. 2f/g vs h (col1: minor differences in statistics, black and white picture shown). Same applies for 3b/c!

6. In terms of proliferation in Figure 2, more analyses than Ki67 are needed (cyclins, PCNA, Western blots etc.).

7. Suppl. Fig. 4: Provide data for liver/bodyweight ration! The interpretation of the findings in the present form are not valid, this could have any reason.

8. The authors state that despite their molecular markers, liver/body weight ration does not change after PH. This definitely needs a much more thorough examination than provided. Are there more cells or not? Mitotic figures? Is there cell shrinkage in the knockouts? More data are needed; otherwise the significance of the effect suggested by the authors is questionable.

In the present form, the data are more appropriate for a more hepatology-focussed journal. Alternatively, the authors should invest much more to elaborate the molecular mechanisms behind their interesting phenotypes.

1st Revision - authors' response 17 November 2014

Response to Editor's and Reviewers' comments

Editor

COMMENT: As you will see, the Reviewers find merits in your manuscript but raise significant issues. I will not well into much detail, as their comments are thorough. I would like, however, to highlight a few main points. Firstly, all three Reviewers clearly agree that your manuscript suffers from a lack of sufficient mechanistic insight in general and specifically into the mode of action of endosialin (EN). This is clearly a major and shared item of dissatisfaction that I'm afraid we agree upon. There are also specific additional concerns from each Reviewer. Reviewer 1 contends that the effects of EN knockout are modest and suggests that additional approaches should be employed to confirm the conclusions including blinded pathology grading, liver function tests, etc. Reviewer 2 notes that your conclusions on the role of EN are based on a whole body knockout model and that a conditional knockout would be required to draw firm conclusions regarding the specific involvement of HCSs. Reviewer 3 is especially concerned that the molecular link between EN knockout and IGF2 is not demonstrated. This Reviewer also notes that it would be of great interest to test EN targeting drugs in vivo. Finally, s/he mentions that some conclusions are not sufficiently supported by the data. In conclusion, while publication of the paper cannot be considered at this stage, given the potential interest of your findings and the fact that the Reviewers, although highly critical, did not call for outright rejection at this time, we have decided to give you the opportunity to address the above concerns. We are thus prepared to consider a substantially revised submission, with the understanding that the Reviewers' concerns must be addressed with **additional experimental data where appropriate and that acceptance of the manuscript will entail a second round of review.** While Reviewer 2's point that a conditional knockout would be better is well taken, we appreciate that to carry out such experimentation would be perhaps further reaching. Nevertheless, in addition to addressing the other concerns, I would encourage you to tackle the specificity of action issue raised by Reviewer 2 as far as realistically possible. In addition, if you could show, as Reviewer 3 suggests, the efficacy of EN-targeted drugs in vivo, this would increase the translational impact of your work while decreasing the level of mechanistic insight required.

RESPONSE: We sincerely appreciate the editor's and reviewers' overall positive assessment of our work. When submitting the work, we were fully aware of the mechanistic limitations of the manuscript. Yet, we submitted the manuscript in the spirit of the "*report*" category of EMBO Mol Med manuscripts *(…concise manuscripts that highlight a specific finding with high impact on the field of molecular medicine…*) considering the conceptually novel and provocative finding of hepatic stellate cell-expressed Endosialin acting as a balancer of hepatocyte proliferation and liver fibrosis during liver damage. We most sincerely appreciate that the reviewers have acknowledged and praised this conceptual advance and we would like to thank them and the editors for the invitation to submit a properly revised manuscript taking into consideration the very constructive comments made by the reviewers.

A wholehearted effort has been made to address in full the many reviewers' comments. In doing so, we believe that the manuscript has substantially matured to now make a very comprehensive and concise contribution to the journal. Please see our response to the individual reviewers' comments below for our detailed answers on how we have through additional experiments and/or editorial revisions advanced the manuscript. In brief, the key points summarized by the editor, have been addressed as follows:

- MOA of Endosialin: Admittedly, the detailed mechanism of action of Endosialin remains to be unraveled. As a C-type lectin, it essentially continues to be an orphan receptor making it really difficult to yield detailed insights into its mechanism of action (we would, by analogy, just like to refer the editor and the reviewers to the hitherto poorly defined MOA of the selectins [even after 20 years of research]). Nevertheless, we believe that the experiments suggested by the reviewers have helped to shed some insights into Endosialin function expressed by HSC. Among others, the experiments suggest a paracrine effect of Endosialin on other liver cell populations (notably hepatocytes and endothelial cells), which serve as source of hepatic acting mitogens and an

autocrine effect of Endosialin in shaping the fibrotic HSC phenotype. Moreover, we provide some evidence for a direct pro-mitogenic role of IGF2, which is upregulated in hepatectomy-challenged Endosialin-deficient mice. Please see the detailed response to the reviewers' comments for all additional experiments added in the revised manuscript.

- Intensity of reported phenotype and blinded morphological analysis: The most exciting aspect of the manuscript undoubtedly is the association of a specific HSC cell surface molecule – Endosialin – towards the balancing of liver regeneration and liver fibrosis during liver damage. Beyond this conceptual advance, we consider the reported findings also quantitatively quite substantial. Endosialin-deficiency leads to a 30% reduction of the total amount of fibrotic tissue including reduced perisinusoidal fibrosis and a significantly reduced number of complete porto-portal septa, which can be considered a rather profound phenotype. Concerning the issue of blinded analyses of all pathology specimens (archive and experimental), it comes without saying that all experiments have been pursued in a blinded fashion. The first author (C.M.) and two of the co-authors (T.L., P.S.) are board-certified pathologists with a long-standing record of liver biopsy interpretation, who have performed all analyses in line with the state-of-the-art. We did not include a Metavir analysis of the liver sections in the original submission as this scoring system is only well established for chronic viral hepatitis in liver core biopsies (notably hepatitis C). Following the reviewers' advice, we have included such analysis in the revised manuscript (Fig. 2e). Furthermore, we performed additional liver function tests and correlation analyses with collagen deposition findings that support the histological, immunhistochemical and biochemical results (Fig. 2m and Suppl. Fig. 10).
- Global KO vs. cell type-specific KO: One of the reviewers criticized that the study was based on a global knockout rather than a HSC-specific KO. We can not quite follow this line of argumentation: Data presented in this study and data previously published by us and others have solidly established Endosialin as cell surface receptor exclusively expressed by selected activated cell types of the mesenchymal lineage, most notably MSC, pericytes, and tumor-associated myofibroblasts (MacFadyen et al, Febs Lett, 2005; MacFadyen et al, Gene Expr Pattern, 2007; Christian et al., Am J Pathol, 2008). We have in the present study quite extensively demonstrated the HSC and myofibroblast-specific expression of Endosialin. As such, an HSC-specific KO of Endosialin would not add additional information to the reported data.
- Link between Endosialin and IGF2: Reviewer 3 had rightfully pointed to the controversial literature on the role of IGF2 in controlling hepatocyte proliferation with some papers arguing for an associative upregulation, whereas other papers have reported a causal role of IGF2 in controlling hepatocyte proliferation. In advancing the manuscript, we have performed additional experiments supporting a role of IGF2 as regulator of hepatocyte proliferation. Details of these experiments (Western blots, cell culture experiments, in vivo experiments) are summarized in our response to Reviewer 3 (see also revised Fig. 3g-m).
- Experiments with EN-targeting drugs in vivo: An Endosialin-targeting antibody is in clinical development in the field of oncology. Consequently, we would gladly like to perform Endosialin antibody-neutralizing antibody experiments in our manipulatory mouse experiments (n.b.: Our recently published Angiopoietin-2 liver regeneration paper has greatly benefitted from the fact that we obtained exactly the same findings in KO mice as with WT mice treated with a neutralizing AB [Hu et al., Science, 2014]). Unfortunately, such Endosialin antibody experiments are not doable at this time, because the above-mentioned Endosialin antibody MORAb-004, which is tested in clinical trials, is a human Endosialin-specific antibody that does not crossreact with mouse Endosialin. We have so far failed to generate a mouse-specific Endosialin neutralizing antibody. We are also in contact with the MORAb-004 company Morphotek, which similarly has not yet been able to generate mouse-specific or mouse-crossreactive function-blocking Endosialin antibodies. As a result, we can at present only refer to genetic loss-of-function experiment, which, however, may be considered highly predictive of antibody blocking experiments.

In conclusion, we believe that the reviewers' comments have very much helped to advance the manuscript to now make it a solid and definite contribution to the journal. Beyond its 3 comprehensive main figures (with >10 display items per figure), the manuscript has now grown to 21 online supplementary figures. As outlined above, we believe that the manuscript is perfectly in line with the "report" format of the journal. Along the same lines, we would also like to address in this introductory comment the suggestion of Reviewer 3 that the manuscript may be more suitable for a hepatology journal. We have submitted our manuscript to EMBO Mol Med and not to a hepatology journal (even though these may have a higher IF), because we favor the focus on the conceptual advance (proliferation vs. fibrosis), which we consider of major interest for the broader molecular medicine community.

Reviewer 1

COMMENT 1: This is a fascinating and novel study that identifies endosialin (EN) to be induced in hepatic stellate cells during liver injury and to mediate paracrine inhibition of hepatocyte regeneration in the injured liver. A candidate approach suggested that IGF2 is an EN-regulated factor that may be responsible for the observed negative impact of EN on regeneration, although this idea was not formally tested. Overall the work addresses an important emerging theme of the interplay between regenerative and fibrogenic mechanisms in the injured liver and supports the concept that stimulating hepatocyte regeneration may prevent fibrosis. Moreover, the study supports recent published evidence that activated liver myofibroblasts may actively repress regeneration, although it is important to note that these cells have also been proposed to be stimulators of hepatocyte proliferation. The strengths of the study are: (1) the use of well-established liver injury and regeneration models to demonstrate up regulation of EN in the injured liver and combined use of EN knockout mice to show that the lectin plays a pro-fibrogenic and anti-regenerative role, and (2) the application of histological, immunohistochemical and biochemical measurements to demonstrate effects of EN knockout on liver physiology and pathology.

RESPONSE 1: We sincerely appreciate the Reviewer's overall positive assessment of our work and for sharing our excitement about the proliferation vs. fibrosis balancing concept.

COMMENT 2: The effects of EN knockout on fibrosis appears modest and many of the measurements are either at the mRNA level or from morphometric analysis of stained liver sections. Ideally, changes in the expression of key proteins such as aSMA should be shown by Western blotting. Given the modest effect of EN knockout on fibrosis it would be preferred that the authors carry out a blinded pathology grading of liver damage and fibrosis using a well-accepted grading systems (e.g. Metavir).

RESPONSE 2: As eluted to in the Response to the Editor's comment (see above), we believe that the study shows some effects that can be considered quite substantial for this kind of in vivo manipulatory experiments (approx. 30% reduction of the total amount of fibrotic tissue including reduced perisinusoidal fibrosis and a significantly reduced number of complete porto-portal septa). Following the Reviewer's advice, we have confirmed and extended the qPCR analyses by Western blots analyses for PDGFR beta, collagen I, αSMA after 6 weeks of CCl_4 treatment (revised Fig. 2i and Suppl. Fig. 8). The morphological analyses have all been performed by board-certified pathologists in a blinded fashion (see also response to Editor's comment). Lastly, we have followed the Reviewer's suggestion to include a METAVIR score analysis (Fig. 2e).

COMMENT 3: The authors should examine the effects of EN knockout on serum liver function tests (ALT, AST, ALP). If as they suggest EN knockout is hepatoprotective then at least in the chronic CCl_4 model this should be reflected in improved LFTs. It would also be important to look at LFTs in the acute CCl4 model at an early time point (6-12hrs) in order to rule out a direct effect of EN on the CCL4-induced liver damage mechanism.

RESPONSE 3: Following the Reviewer's suggestion, we have included additional liver function tests (Fig. 2m and Suppl. Fig. 10) showing a remarkable improvement of liver serum enzymes in EN-deficient mice after 6 weeks of CCl_4 treatment. As suggested, we also performed acute CCl_4 toxicity studies in wild type and Endosialin-deficient mice. There was no difference in liver enzymes in blood serum and liver histology (H&E and silver stains) after 9 hrs of CCl_4 treatment ruling out a direct effect of EN on the CCl_4-induced liver damage mechanism (Suppl. Fig. 5).

COMMENT 4: The weakest part of the study is its mechanistic component, i.e. how is EN expressed on hepatic myofibroblasts modulating hepatocyte regeneration. The authors identify IGF2 as a potential mediator but do not formally test this idea either in vivo or in vitro. Without formally testing if IGF2 mediates the effects of EN this important component of the study is superficial at best. It is also difficult without this mechanistic information to envisage how and why EN expressed on myofibroblasts should regulate hepatocyte proliferation. What is the cellular source of the IGF2 and how is regulated by EN are further important questions. Furthermore the candidate approach is limited, ideally the authors should have presented genome-wide data which is now cheap enough and available to all labs.

RESPONSE 4: Given the C-type lectin orphan receptor status of Endosialin, definite data on the molecular MOA are presently difficult to present (see also response to Editor's comment). Yet, we believe that the many additional experiments stimulated by the reviewers' comments have greatly helped to shed mechanistic insights into Endosialin function: Concerning the proliferation vs. fibrosis balance concept, we can now pin down the proliferation phenotype to paracrine-mediated Endosialin effects (Fig. 3j,k,l; Suppl. Fig. 12, 20), whereas the fibrosis findings are due to autocrine functions of Endosialin related to the fibrotic reprogramming of HSC during fibrosis (Fig. 2n). Concerning the role of IGF2 on hepatocytes, we show in the revised manuscript that IGF2 is produced primarily by endothelial cells and hepatocytes (Fig. 3j,k,l; Suppl. Fig. 20) suggesting a paracrine MOA of HSC expressed Endosialin on other cells . Moreover, we could show that IGF2 directly regulates hepatocyte proliferation (Fig. 3m). Concerning genome-wide transcriptomic analyses, we have several years ago already performed array-based analyses of WT and KO mouse embryonic fibroblasts (MEF) and whole liver lysates one day after partial hepatectomy. Unfortunately, the quality of the data from these experiments was limited either due to studying the wrong cell population (MEFs) or starting from a crude lysate (whole liver lysates). Likewise, the limited yield of in vivo purified HSC limited array-based analyses of freshly isolated cells and analyses of in vitro expanded cultured HSC is similarly not particularly instructive. It is for these reasons that we have for the experiments of this manuscript abandoned genome wide screens to focus on a candidate-based approach of known regulators of liver regeneration and fibrosis.

Reviewer 2

COMMENT 1: The evidence is largely based on data derived in a global knockout of endosialin, making it difficult to know how much of the phenotypic alterations are directly due to loss of endosialin in stellate cells themselves. A stellate cell-specific knock out would be required to do this.

RESPONSE 1: As already pointed out in our response to the Editor's comment, we can not quite follow this line of arguments: Data presented in this study and data previously published by us and others have solidly established Endosialin as cell surface receptor exclusively expressed by selected activated cell types of the mesenchymal lineage, most notably MSC, pericytes, and tumor-associated myofibroblasts (MacFadyen et al, Febs Lett, 2005; MacFadyen et al, Gene Expr Pattern, 2007; Christian et al., Am J Pathol, 2008). We have in the present study quite extensively demonstrated the HSC and myofibroblast-specific expression of Endosialin. As such, a HSC-specific KO of Endosialin would not add additional information to the reported data.

COMMENT 2: Assuming loss of stellate cell-derived Endosialin is driving the phenotype, more work is needed to delineate the mechanisms. For example, in what way does loss of Endosialin change stellate cells? Does stellate cell conditioned medium from wild type and knock out stellate cells differentially impact hepatocyte proliferative activity?

RESPONSE 2: Lentiviral knockdown of Endosialin in LX-2 cells (immortalized human hepatic stellate cells) resulted in a strong reduction of fibrogenic markers (αSMA as well as type I and type VI collagen) within 24 hrs in Endosialin-silenced HSC compared to control (Fig. 2n) suggesting an autocrine effect of Endosialin in shaping the HSC phenotype during fibrogenesis. Long-term culture (8 days) of isolated HSC from wildtype and Endosialin-deficient mice yielded morphologically indistinguishable KO and WT cells (Suppl. Fig. 12). Supernatants from these cells elicited no difference in hepatocyte proliferation (Fig. 3l) suggesting a primarily paracrine effect of cell surface-expressed Endosialin on other cell populations, which would be compatible with the identification of endothelial cells and hepatocytes as primary source of IGF2 (Fig. 3jk). We attempted to demonstrate such paracrine effects in direct HSC and EC or hepatocyte co-culture experiments. Yet, such co-culture experiments are technically not feasible due to rather different requirements of the different cell populations.

COMMENT 3: 2 - Assuming loss of stellate cell-derived endosialin is driving the phenotype, more work is needed to delineate the mechanisms: (i) In what way does loss of Endosialin change stellate cells?; (ii) Does stellate cell conditioned medium from wild type and knock out stellate cells differentially impact hepatocyte proliferative activity?, and (iii) does Endosialin (or its deficiency) influence epithelial-to mesenchymal or mesenchymal to epithelial transitions?

RESPONSE 3: For questions (i) and (ii), we kindly refer to RESPONSE 2. Additionally performed qRT-PCR analyses of liver fibrosis samples after 2, 4 and 6 weeks of CCl_4 treatment did not reveal significant differences in epithelial-to-mesenchymal transition markers (Suppl. Fig. 11).

Reviewer 3

COMMENT 1: In their work, the authors addressed the possible function of Endosialin, a c-type lectin, in models of liver disease. The authors provide evidence that Endosialin, which seems to be expressed mainly by hepatic stellate cells, promotes liver fibrosis but in turn suppresses proliferation of hepatocytes, making it an attractive candidate for pharmacological targeting in chronic liver disease. From a technical point of view, these are clean and solid data. My main concern, however, is that the whole manuscript appears quite preliminary, lacking a conclusive molecular explanation for the potentially very interesting effect of Endosialin-knockout in these models.

RESPONSE 1: We mostly agree with the reviewer and share his/her assessment of our work. Clearly, this is not a mechanistically complete story. That's why we felt that it would be most suitable for EMBO Mol Med's "report" format (please see also our response to the Editor comment above). Endosialin is an orphan receptor C-type lectin. It is a biologically fascinating molecule, but it will take quite some time to solidly understand its MOA (please see also above analogy comment to the selectins). Nevertheless, despite these limitations, we believe that the revised manuscript sheds beyond an interesting and potentially therapy-relevant KO phenotype in challenge experiments some relevant mechanistic insights into Endosialin functions. As outlined in more detail elsewhere, the experiments proposed by the reviewers have helped to characterize the autocrine effects of Endosialin on the fibrogenic reprogramming of HSCs and its paracrine effects (on hepatocytes and EC) on hepatocyte mitogenic activity, including IGF2.

COMMENT 2: Most importantly, there is no explanation at present how Endosialin (EN) on HSC controls liver fibrosis. I do not see any experiment providing data for this functional question.

RESPONSE 2: Our results from human and murine liver fibrosis samples suggest that Endosialin is a marker of early activated myofibroblasts capable of postponing the activation process. Lentiviral

mediated EN knockdown in human hepatic stellate cells (LX-2) strongly supports this hypothesis in vitro as a 90% knockdown of Endosialin led to a dramatic reduction of fibrogenic and activation markers. The results of thee experiments are included in Fig. 2n of the revised manuscript.

COMMENT 3: The molecular linkage between EN knockout and the supposed target gene IGF-2 is not convincing at all and at this stage precludes publication in EMM. This assumption is solely based on one single RT-PCR. From our experience, IGF-2 is rather a marker of proliferation in liver models (similar to H-19) and is upregulated in proliferating liver cells in most models we looked at. The hypothesis that EN in HSC directly controls IGF-2 (how?) which again is the driving factor for hyper proliferation (how) is not backed up (and to my point of view rather unlikely). A whole new set of experiments is needed from my point of view to provide a functional explanation for these important questions

RESPONSE 3: We agree with reviewer that the literature on the role of IGF2 in the regulation of hepatocyte proliferation is controversial and it has not unambiguously been established if IGF2 is a an associatively upregulated marker of hepatocyte proliferation or a causally involved regulator. Following the Reviewer's suggestion, we have performed additional experiments to assess the sourced of IGF2 and its effects on hepatocytes. We have identified hepatocytes and endothelial cells as source of IGF2 (Fig. 3j,k) – suggesting a paracrine mode-of-action of HSC expressed Endosialin. Moreover, we confirmed in cellular experiments a stimulating effect of IGF2 on hepatocyte proliferation (Fig. 3m). These experiments were accompanied by phosphorylation of the IGFR-1and IRS1 (Fig. 3h) as well as phosphorylation of downstream signaling pathways, such as AKT and ERK (Fig. 3j).

COMMENT 4: Given the clinical focus of EMM, it would be interesting if the authors tested one of the available drugs targeting EN in their mouse model of liver fibrosis.

RESPONSE 4: As already outlined in our response to the Editor comment in some detail, we would love to do such experiments. Yet, they are presently not feasible because there are no suitable mouse Endosialin reagents available. As such, it may on the one hand be considered a limitation that our work is just based on a genetic model. In turn, we believe that the genetic model will be quite predictive of antibody blocking experiments (e.g., as we have recently shown in our liver Angiopoietin-2 regeneration work [Hu et al., Science, 2014]).

COMMENT 5: The results part starts with a quite confusing section. Clearly distinguish introduction and results.

RESPONSE 5: We agree with the reviewer and have re-drafted the manuscript accordingly.

COMMENT 6: I understood that HSC were separated by density gradient centrifugation. This method is inferior to more advanced techniques, e.g. combinations with FACS-based cell sorting in terms of purity. It might make sense to check expression data in pure primary HSC.

RESPONSE 6: FACS sorting is nowadays the most widely employed method of obtaining pure cell population. Accordingly, we routinely employ FACS sorting towards this end. Yet, in the particular case of hepatic stellate cells, the apparently old-fashioned technique of density gradient centrifugation (DGC) has a number of distinct advantages, for which reason we actually consider it superior to FACS sorting. First, purity is really not an issue and the samples analyzed in our experiments are of very high purity (e.g., compare Suppl. Fig. 5 of Hu et al. [Science, 2014] which establishes by differential marker analysis the purity of the different liver populations studied in our laboratory). Second, DGC allows the separation HSC subpopulations with distinct activation characteristics. This enabled us to trace HSC Endosialin expression to the activated 8.25% DGC layer as shown in Fig. 2c.

COMMENT 7: The Immunostainings for SMA and EN are not completely clear to me. Are all SMA+ cells EM+, e.g. in cirrhosis? Are also other cells positive? Provide double stainings.

RESPONSE 7: As shown in Fig. 1i-n as well as in Suppl. Fig. 1, Endosialin-positive cells are partially positive for other (myo-)fibroblast markers such as aSMA or PDGFRb but not for smooth muscle markers such as caldesmon. Together with our findings that Endosialin is predominantly upregulated in developing liver fibrosis, we consider Endosialin as a marker of early activated (myo-)fibroblasts. The immunhistochemical analysis solidly established that no other cells than myofibroblasts and pericytes are EN+. Even though we have already several years ago unambiguously shown that Endisialin is not expressed by endothelial cells (Christian et al., Am J Pathol, 2008), we have performed additional double stainings for CD31 and Endosialin in cirrhotic livers which provided no evidence whatsoever for non-HSC and non-myofibroblast expression of Endosialin (Suppl. Fig. 2).

COMMENT 8: The statement on equal liver damage in both experimental groups is based solely on cleavage of Casp-3. This is clearly not sufficient, since other forms of cell death might be much more relevant in CCl_4 than apoptosis. Provide serum AST, ALT, GLDH data and necrosis analysis.

RESPONSE 8: Following the Reviewer's suggestion, we have included additional liver function tests and necrosis analysis of HE-stained paraffin slides in the revised manuscript (Fig. 2e, m; Suppl. Fig. 10).

COMMENT 8: Are all shown figures representative for the statistics? This appears critical in Fig. 2f/g vs h (col1: minor differences in statistics, black and white picture shown). Same applies for 3b/c!

RESPONSE 8: We carefully selected representative images for the original submission of our manuscript to most appropriately match the quantitative analyses. Acknowledging the Reviewer's comment, we have in the revised manuscript one more time meticulously matched representative images and quantitative analyses and made few changes to absolutely avoid that the optical impression may not perfectly match the quantitative data. Accordingly, Figg. 2f, g and 3d, e [originally 3b,c) have been replaced.

COMMENT 9: In terms of proliferation in Fig. 2, more analyses than Ki67 are needed (cyclins, PCNA, Western blots etc.).

RESPONSE 9: As suggested, we have included additional IHC stainings (PCNA, Cyclin D1) confirming the Ki67 findings (Suppl Fig. 13). Western blot analyses for PCNA (please see below) showed only minor differences most likely due to the fact that the major cell type proliferating in the setting of CCl_4-induced liver fibrosis are recruited immune cells and not necessarily the hepatocytes (marked with arrows in image on the left).

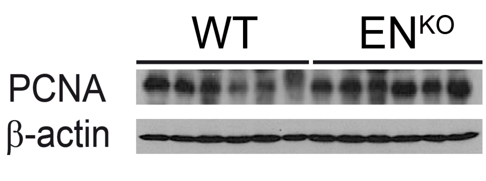

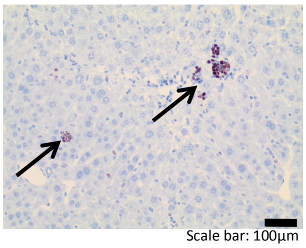

COMMENT 10: Suppl. Fig. 4: Provide data for liver/body weight ration! The interpretation of the findings in the present form are not valid, this could have any reason.

RESPONSE 10: We do not quite understand the reviewer´s question. Liver to body weight ratios had quantitatively been given for all experimental groups in Suppl. Fig. 4 (now Suppl. Fig. 17e). We can gladly forward the raw data that have led to this figure for review, but this would probably not answer the Reviewer's question. We do understand the apparent confusion why the hepatocyte proliferative phenotype did not translate into significant difference in liver to body weight ratio. Yet, this is not an unusual finding and can be observed in quite a few publications. In order to further address this enigma, we have (also in consideration of the Reviewer's comment #11) performed

additional morphometric analyses. These revealed that EN knockout mice had following 2/3 PHx during the peak of hepatocyte proliferation (day 2) higher hepatocyte proliferation. Yet, these cells were smaller in size than hepatocytes in WT PHx experiments (Suppl .Fig. 17a, b). Knockout animals additionally showed a significantly increased mitotic rate 2 days after PHx (Fig. 3f), whereas the number of double nuclei did not differ between knockout and wildtype animals (Suppl. Fig. 17c). Interestingly, the total number of hepatocytes/HPF was almost identical and lower in both groups one day after hepatectomy compared to hepatocytes two days after PHx (Suppl. Fig. 17d). We therefore assume that physiological hepatocyte hypertrophy (e.g., Miyaoka Y et al., Curr Biol, 2012) contributes to liver size and weight in the initial phase of liver regeneration which was similarly initiated in both wildtype and knockout animals. The reduced size of hepatocytes during the proliferative phase of liver regeneration in KO mice would then equalize the gain of weight (and mass) achieved by the observed hyperproliferation.

COMMENT 11: The authors state that despite their molecular markers, liver/body weight ration does not change after PH. This definitely needs a much more thorough examination than provided. Are there more cells or not? Mitotic figures? Is there cell shrinkage in the knockouts? More data are needed; otherwise the significance of the effect suggested by the authors is questionable.

RESPONSE 11: This comment relates to the above comment on cell size/proliferative dynamics/liver weight and we would kindly refer th Reviewer to our response to comment 10 in which we have exactly performed the additional morphometric analyses suggested by the reviewer.

COMMENT 12: In the present form, the data are more appropriate for a more hepatology-focussed journal. Alternatively, the authors should invest much more to elaborate the molecular mechanisms behind their interesting phenotypes.

RESPONSE 12: As already pointed out in our response to the Editor's comment, we had submitted our manuscript to EMBO Mol Med and not to a hepatology journal (even though these may have a higher IF), because we favor the focus on the conceptual advance (proliferation vs. fibrosis), which we consider of major interest for the broader molecular medicine community.

2nd Editorial Decision 12 December 2014

Thank you for the submission of your revised manuscript to EMBO Molecular Medicine. We have now received the enclosed reports from the Reviewers that were asked to re-assess it. As you will see while Reviewers 1 and 3 are now globally supportive, Reviewer 2 is quite critical.

Briefly, Reviewer 2 lists a number of serious concerns and expresses numerous doubts on the conclusiveness of your findings. Although I have to admit that some of the issues now raised partially overlap with those expressed by the other two Reviewers in the first round (but who are now satisfied), admittedly these concerns were not raised in his/her initial evaluation. Please note, however, that Reviewer 3, while globally positive, does still express clear concerns with respect to the experimental support provided on the alleged role of IGF2.

After in depth discussion with my colleagues, including with the Chief Editor, we have decided to invite a final revision, although we will not be asking you to provide further experimentation at this point, unless you should have further data that might strengthen your claims.

Specifically, I would encourage you to 1) send me a rebuttal on the points raised by Reviewer 2 (and 3); 2) provide additional supporting data if available and/or amend your text as to avoid overreaching conclusions on the various points mentioned, including IGF2 and in general to better discuss the limitations of the study and 3) provide statistical analysis where missing (e.g. Fig 1 and Fig. 3).

I am willing to make an Editorial decision on your final, revised version, provided the issues raised are dealt with as mentioned above.

Please also carefully consider the following final Editorial amendments/requests to be include in your revision:

1) As per our Author Guidelines, the description of all reported data that includes statistical testing must state the name of the statistical test used to generate error bars and P values, the number (n) of independent experiments underlying each data point (not replicate measures of one sample), and the actual P value for each test (not merely 'significant' or 'P < 0.05').

2) Please improve the quality of the figures provided in the supplementary information file. Currently we see a grey shading/blurring around some of the panels, e.g. in SF. 4, 6 and 7. Also, a reference to the table appears to be missing (probably it should be in the supplementary information itself?)

3) Please upload separate manuscript (word doc) and figure files (one per figure).

4) We are now encouraging the publication of source data, particularly for electrophoretic gels and blots, with the aim of making primary data more accessible and transparent to the reader. Would you be willing to provide a PDF file per figure that contains the original, uncropped and unprocessed scans of all or at least the key gels used in the manuscript? The PDF files should be labeled with the appropriate figure/panel number, and should have molecular weight markers; further annotation may be useful but is not essential. The PDF files will be published online with the article as supplementary "Source Data" files. If you have any questions regarding this just contact me.

5) Every published paper now includes a 'Synopsis' to further enhance discoverability. Synopses are displayed on the journal webpage and are freely accessible to all readers. They include a short standfirst (to be written by the editor) as well as 2-5 one sentence bullet points that summarise the paper (to be written by the author). Please provide the short list of bullet points that summarise the key NEW findings. The bullet points should be designed to be complementary to the abstract - i.e. not repeat the same text. We encourage inclusion of key acronyms and quantitative information. Please use the passive voice. Please attach these in a separate file or send them by email, we will incorporate them accordingly.

I look forward to seeing a revised form of your manuscript as soon as possible.

***** Reviewer's comments *****

Referee #1 (Comments on Novelty/Model System):

Without mechanistic data it is difficult to assess the likely clinical relevance of the work described.

Referee #1 (Remarks):

The authors have satisfactory addressed my comments.

Referee #2 (Remarks):

This is a revised manuscript that investigates the role of endosialin (EN) in regulating liver repair. EN is expressed by hepatic stellate cells (HSC). Evidence is presented to demonstrate that EN expression increases when HSC become MF both in vivo and in vitro. Comparison of responses to acute and chronic toxic liver injury, as well as acute partial hepatectomy, in EN-knockout (KO) mice and WT controls is said to show decreased fibrosis and increased hepatocyte proliferation in the KO. The latter is attributed to increased expression of IGF2 in the EN-KO group. Loss of EN in HSC is said to promote increased expression of IGF2 by some other liver cell type (e.g., liver sinusoidal endothelial cells) and this, in turn, is felt to drive increased hepatocyte proliferation. The authors conclude that HSC EN promotes liver fibrosis, while it suppresses liver regeneration. Thus, they propose that strategies to inhibit EN would provide a treatment to prevent cirrhosis and normalize regeneration.

The authors have done some new experiments and revised the manuscript to address concerns that the previous reviewers raised about insufficient mechanistic information in the initial manuscript. However, in my view, there are still important limitations in the revision.

My specific concerns follow:
Fig 1: Fig 1a-c demonstrate that HSC express EN. No error bars or statistic are provided in either Fig 1a-1b. This is necessary to assure that the findings were reproducible. F1g 1a also indicates that LSECs express EN. However, the text repeatedly emphasizes that EN is specifically expressed by HSC (and thus, HSC-targeted approaches to delete EN are not necessary to tease out EN effects on liver repair). Please clarify.

Fig 2: Figs 2a- b demonstrate that the kinetics of EN induction closely parallel those of aSMA. Other groups have reported that HSC-derived myofibroblasts (MF) are marked by aSMA and constitute the major source of collagen during liver fibrosis. Consistent with those data, Fig 2d demonstrates a progressive rise in EN during liver fibrosis progression during CCL4-induced liver injury. Fig 2c demonstrates that EN is expressed "early" during HSC activation. However, data in Fig 2e and 2h indicate that fibrosis and collagen gene expression in EN-KO mice is only less than in WT mice after 6 weeks of CCL4 injury. Given that Fig 2l and 2n demonstrate fairly robust (~50% reduction) and consistent decreases in aSMA, desmin, and collagen when EN is deleted, how is this "lag" between change in EN and the relatively minor change in collagen/fibrosis (<20% decrease) explained in the EN-KO mice? In contrast, data shown in Fig 2m indicate that EN-KO mice had significantly less liver injury (>50% reductions in AST, ALT) than WT mice at the 6 week time point. Was the reduced liver injury in the EN-KO group a cause or a consequence of the decreased myofibroblastic HSC in that group?
Fig 3: The photomics in Fig 3a do not reflect the graphs in Fig 3b. To my eye, Ki67 staining seems similar at each of the time points in the two groups while the graph indicates that the EN-KO have significantly fewer Ki67-positive cells. On the other hand, Fig 3d-e demonstrate transient (only at 2d) and relatively modest (i.e., about 10%) differences in hepatocyte proliferative activity between EN-KO and WT mice after partial hepatectomy (PH). Thus, the aggregate data in Fig 3a-e would seem to indicate that EN-KO has a relatively mild and very transient effect on hepatocyte

proliferation.
On the other hand, Fig 3f suggests that EN-KO has a more substantial effect on mitosis. This suggests EN deletion might impact hepatocyte cell cycle progression. However, the Supplemental PNCA and cyclin D1 data do not clarify this issue.
The remainder of Fig 3 attempts to define a mechanism to explain the modest, transient effects of EN deletion on hepatocyte proliferation. This is attributed to IGF2 based on data indicated transcripts of this gene are increased in whole liver in the knockouts (Fig 3g). However, no direct evidence is presented to demonstrate that increased IGF2 is, in fact, the cause for increased hepatocyte DNA synthesis in the EN-KO mice. Proof of this concept would require evidence that inhibiting IGF2 prevents the increase in hepatocyte DNA synthesis that occurs in the EN-KO mice. Data about differences in whole liver phosphor-IGFR1 and phosphor-AKT/ERK (Fig 3h) do not suffice to prove that IGF2 is a) responsible for any of these differences or b) caused increased IGFR1 signaling specifically in hepatocytes. Simply showing that recombinant IGF2 promotes proliferation of cultured hepatocytes is not sufficient to prove that increased IGF2 is the basis for the phenotype observed in the EN-KO mice.
Data in Fig 3j is interpreted as showing that LSECs are a key source of IGF2 in the EN-KO mice. However, the figure does not indicate whether these differences in mRNA expression are statistically significant - are they?
The text and supplemental data indicate that EN-KO HSC generate something that promotes LSECs to increase their production of IGF2. However, no experimental evidence is presented to substantiate this claim. Does immunohistochemistry demonstrate increased IGF2 in resident LSECS of EN-KO mice? Do isolated LSECs from EN-KO mice express more IGF2 when analyzed by FACS or Western blot? Does conditioned medium from EN-KO HSC stimulate LSECs from EN-KO or WT mice to produce IGF2? If direct cell-cell contact is necessary for this to occur (as the authors suggest), does conditioned medium from co-cultures of EN-KO HSC and LSECs have more IGF2 than conditioned medium from co-cultures of WT HSC and LSECs?
Finally, many cell types express receptors for IGF2, including HSC themselves. Does IGF2 influence the transdifferentiation of HSC into aSMA-positive, fibrogenic myofibroblasts?

Referee #3 (Comments on Novelty/Model System):

All three Referees have initially agreed that the paper is lacking mechanistic insight into the shown phenotype. Reading now all referees' comments again, I would probably not have sent it out for Revision, given this fundamental critics.

However, the authors have spent significant efforts on this resubmission, which is acknoledged by this Referee. I disagree with the authors that a lack of mechanistic insights justifies publication in EMM "in the Spirit of a Report". To my understanding, a Report should highlight a clear cut, less complex mechanism, rather than a pure phenotype. Also, I am not yet completely convinced on IGF2 in this process, but the authors provided some more data on this.

Together, I agree with publication, mainly based on the significant efforts spent on this Revision.

2nd Revision received 07 January 2015

EMM-2014-04246-V2 - Response to Editor's and Reviewers' comments

EDITOR'S COMMENT

GENERAL COMMENT: Thank you for the submission of your revised manuscript to EMBO Molecular Medicine. We have now received the enclosed reports from the Reviewers that were asked to re-assess it. As you will see while Reviewers 1 and 3 are now globally supportive, Reviewer 2 is quite critical. Briefly, Reviewer 2 lists a number of serious concerns and expresses numerous doubts on the conclusiveness of your findings. Although I have to admit that some of the issues now raised partially overlap with those expressed by the other two Reviewers in the first round (but who are now satisfied), admittedly these concerns were not raised in his/her initial evaluation. Please note, however, that Reviewer 3, while globally positive, does still express clear concerns with respect to the experimental support provided on the alleged role of IGF2.

After in depth discussion with my colleagues, including with the Chief Editor, we have decided to invite a final revision, although we will not be asking you to provide further experimentation at this point, unless you should have further data that might strengthen your claims. Specifically, I would encourage you to 1) send me a rebuttal on the points raised by Reviewer 2 (and 3); 2) provide additional supporting data if available and/or amend your text as to avoid overreaching conclusions on the various points mentioned, including IGF2 and in general to better discuss the limitations of the study and 3) provide statistical analysis where missing (e.g. Fig 1 and Fig. 3).

RESPONSE: We are very pleased by the overall positive feedback of reviewer #1 and #3 and really appreciate the praise for the effort we made in the revision process to substantially advance our manuscript. As we have already mentioned in our first rebuttal letter, we were still not able to fully clarify the underlying mechanism. For that reason we decided to stay with the "report" format. Nevertheless, we believe that our findings will now make a strong and solid contribution to EMBO MOL MED. As for the specific points raised above, we have 1) drafted this rebuttal letter to address all reviewers' comments, 2.) have removed all overreaching conclusions from the manuscript, most notably as it relates to IGF2 (in fact, the comprehensive combined 'Results and Discussion' section is written very factual and leaves little room for extensive interpretation of the data), and 3.) provided all the missing statistical analyses.

SPECIFIC COMMENT 1: As per our Author Guidelines, the description of all reported data that includes statistical testing must state the name of the statistical test used to generate error bars and P values, the number (n) of independent experiments underlying each data point (not replicate measures of one sample), and the actual P value for each test (not merely 'significant' or 'P < 0.05').

RESPONSE: This has been done.

SPECIFIC COMMENT 2: Please improve the quality of the figures provided in the supplementary information file. Currently we see a grey shading/blurring around some of the

panels, e.g. in SF. 4, 6 and 7. Also, a reference to the table appears to be missing (probably it should be in the supplementary information itself?).

RESPONSE: This has been done. The supplementary file now has a size of almost 30 MB, but the higher resolution has improved the visual quality substantially.

SPECIFIC COMMENT 3: Please upload separate manuscript (word doc) and figure files (one per figure).

RESPONSE: This has been done.

SPECIFIC COMMENT 4: We are now encouraging the publication of source data, particularly for electrophoretic gels and blots, with the aim of making primary data more accessible and transparent to the reader. Would you be willing to provide a PDF file per figure that contains the original, uncropped and unprocessed scans of all or at least the key gels used in the manuscript? The PDF files should be labeled with the appropriate figure/panel number, and should have molecular weight markers; further annotation may be useful but is not essential. The PDF files will be published online with the article as supplementary "Source Data" files. If you have any questions regarding this just contact me.

RESPONSE: We have included the original blots shown in the figures of the manuscript as PDF source file.

SPECIFIC COMMENT 5: Every published paper now includes a 'Synopsis' to further enhance discoverability. Synopses are displayed on the journal webpage and are freely accessible to all readers. They include a short standfirst (to be written by the editor) as well as 2-5 one sentence bullet points that summarise the paper (to be written by the author). Please provide the short list of bullet points that summarise the key NEW findings. The bullet points should be designed to be complementary to the abstract - i.e. not repeat the same text. We encourage inclusion of key acronyms and quantitative information. Please use the passive voice. Please attach these in a separate file or send them by email, we will incorporate them accordingly

RESPONSE: We have drafted a synopsis and sent this by email to the editorial office.

REVIEWER 1

COMMENT: Without mechanistic data it is difficult to assess the likely clinical relevance of the work described. The authors have satisfactory addressed my comments.

RESPONSE: We again thank the reviewer for the very constructive comments and critical concerns that helped to greatly improve the manuscript. Although not being able to fully unravel the underlying molecular mechanisms in detail, we are confident that the experimental data in a genetic mouse model supported by human pathology-based expression profiling data will have clinical impact and may pave the way towards novel therapeutic strategies for liver fibrosis.

REVIEWER 2

GENERAL COMMENT: This is a revised manuscript that investigates the role of Endosialin (EN) in regulating liver repair. EN is expressed by hepatic stellate cells (HSC). Evidence is presented to demonstrate that EN expression increases when HSC become MF both in vivo and in vitro. Comparison of responses to acute and chronic toxic liver injury, as well as acute partial hepatectomy, in EN-knockout (KO) mice and WT controls is said to show decreased fibrosis and increased hepatocyte proliferation in the KO. The latter is attributed to increased expression of IGF2 in the EN-KO group. Loss of EN in HSC is said to promote increased expression of IGF2 by some other liver cell type (e.g., liver sinusoidal endothelial cells) and this, in turn, is felt to drive increased hepatocyte proliferation. The authors conclude that HSC EN promotes liver fibrosis, while it suppresses liver regeneration. Thus, they propose that strategies to inhibit EN would provide a treatment to prevent cirrhosis and normalize regeneration. The authors have done some new experiments and revised the manuscript to address concerns that the previous reviewers raised about insufficient mechanistic information in the initial manuscript. However, in my view, there are still important limitations in the revision.

RESPONSE: We sincerely appreciate the Reviewer's appreciation of our work. As outlined in more detail below, we have addressed the Reviewer's comments and concerns and edited the manuscript whenever necessary or appropriate.

COMMENT 2: Fig 1a-c demonstrate that HSC express EN. No error bars or statistic are provided in either Fig 1a-1b. This is necessary to assure that the findings were reproducible. F1a also indicates that LSECs express EN. However, the text repeatedly emphasizes that EN is specifically expressed by HSC (and thus, HSC-targeted approaches to delete EN are not necessary to tease out EN effects on liver repair). Please clarify.

RESPONSE: LSEC isolation was performed on unchallenged wildtype mice. Purity of the LSEC preparations was always >90%. Despite such a high purity, we cannot fully exclude a minor contamination by HSC and other EN+ cells such as myofibroblasts. The small expression bar in Fig. 1a is consequently interpreted to reflect the minor impurity in the isolated LSEC. To clarify this issue, we added q-rt PCR data from LX-2 cells (immortalized human HSC) and immunohistochemical double staining of human livers (Suppl. Fig. 2) for Endosialin and CD31, which clearly show that Endosialin expression does not overlap with CD31+ EC. The experimental data (and also our previously published work, e.g., Christian et al, Am J Path, 2008) conclusively exclude LSEC expression of Endosialin.
Following the advice of the Reviewer, we included error bars in Fig. 1a and 1b.

COMMENT 2: Figs 2a- b demonstrate that the kinetics of EN induction closely parallel those of αSMA. Other groups have reported that HSC-derived myofibroblasts (MF) are marked by αSMA and constitute the major source of collagen during liver fibrosis. Consistent with those data, Fig 2d demonstrates a progressive rise in EN during liver fibrosis progression during CCL_4-induced liver injury. Fig 2c demonstrates that EN is expressed "early" during HSC activation. However, data in Fig 2e and 2h indicate that fibrosis and collagen gene expression in EN-KO mice is only less than in WT mice after 6 weeks of CCL_4 injury. Given that Fig 2l and 2n demonstrate fairly robust (~50% reduction) and consistent decreases in αSMA, desmin, and collagen when EN is deleted, how is this "lag" between change in EN

and the relatively minor change in collagen/fibrosis (<20% decrease) explained in the EN-KO mice?

RESPONSE: We believe that the presented data are fully consistent within the study and also with the published literature. What the Reviewer tries to point out as apparent discrepancies are really very different experiments: Fig. 2a-c are acute (single) high dose CCL_4 experiments. These show that Endosialin is in this acute setting indeed activated rapidly and transiently – essentially phenocopying the dynamics of αSMA expression. In contrast, Fig. 2d-m are lower dose (repeated) CCL_4 experiments to study chronic changes. These are associated with a gradual increase of Endosialin expression. Concomitantly, the consequences of genetic Endosialin deletion become quantitatively apparent only after 6 weeks. As such, there is no "lag" between Endosialin expression and fibrosis, but the chronic situation only builds up over time. Lastly, Fig. 2l (mouse experiment) and Fig. 2n (tissue culture) should not be confused – these are rather different experiments. Yet, all data show conclusively (*in vivo* or *in vitro*) that Endosialin knockout/silencing leads to reduced expression of fibrosis-related transcripts.

COMMENT 3: In contrast, data shown in Fig 2m indicate that EN-KO mice had significantly less liver injury (50% reductions in AST, ALT) than WT mice at the 6 week time point. Was the reduced liver injury in the EN-KO group a cause or a consequence of the decreased myofibroblastic HSC in that group?

RESPONSE: Hepatocyte damage leads to elevated levels of ALT and AST in the serum. Yet, these biomarker readouts of liver damage do not allow an interpretation of the cause of liver damage. Given that Endosialin is exclusively expressed by HSC and myofibroblasts and not by hepatocytes, we obviously interpret the reduced levels of liver enzymes reflecting reduced liver injury as a consequence of the altered HSC/myofibroblast behavior in the EN-KO group.

COMMENT 4: Fig 3: The photomics in Fig 3a do not reflect the graphs in Fig 3b. To my eye, Ki67 staining seems similar at each of the time points in the two groups while the graph indicates that the EN-KO have significantly fewer Ki67-positive cells. On the other hand, Fig 3d-e demonstrate transient (only at 2d) and relatively modest (i.e., about 10%) differences in hepatocyte proliferative activity between EN-KO and WT mice after partial hepatectomy (PH). Thus, the aggregate data in Fig 3a-e would seem to indicate that EN-KO has a relatively mild and very transient effect on hepatocyte proliferation. On the other hand, Fig 3f suggests that EN-KO has a more substantial effect on mitosis. This suggests EN deletion might impact hepatocyte cell cycle progression. However, the Supplemental PCNA and cyclin D1 data do not clarify this issue.

RESPONSE: As in comment 3, different types of experiments should not be compared or mixed up: Fig. 3a-b show proliferation data of chronic CCL_4 fibrosis experiments, whereas Fig. 3c-e show proliferation data of acute hepatectomy experiments. The representative images shown in Fig. 3a are perfectly compatible with the quantitation shown in Fig. 3b. Yet, we agree with the reviewer that the images may be a little too small to really appreciate these images well. We have consequently replaced the images with corresponding higher magnification images. Quantitatively, the effects shown in Fig. 3b are quite substantial, reflecting the prominent role of the HSC/myofibroblast response during chronic challenge.

As for Fig. 3c-e, the effects are indeed smaller (approx. 20% [19.8% to be exact] and not 10% as stated by the reviewer. Yet, we consider even this smaller difference biologically quite relevant and surprising considering the overall less prominent involvement of the HSC/myofibroblast compartment during these short-term hepatectomy experiments.
Concerning the effects on mitosis, the mitotic count is an additional readout of proliferation (included during the R1 revision in response to one of the Reviewer's comments). These findings are not contradictory, but perfectly compatible and complementary to the PCNA and Cyclin D1 staining data.

COMMENT 5: The remainder of Fig. 3 attempts to define a mechanism to explain the modest, transient effects of EN deletion on hepatocyte proliferation. This is attributed to IGF2 based on data indicated transcripts of this gene are increased in whole liver in the knockouts (Fig 3g). However, no direct evidence is presented to demonstrate that increased IGF2 is, in fact, the cause for increased hepatocyte DNA synthesis in the EN-KO mice. Proof of this concept would require evidence that inhibiting IGF2 prevents the increase in hepatocyte DNA synthesis that occurs in the EN-KO mice. Data about differences in whole liver phosphor-IGFR1 and phosphor-AKT/ERK (Fig 3h) do not suffice to prove that IGF2 is a) responsible for any of these differences or b) caused increased IGFR1 signaling specifically in hepatocytes.

RESPONSE: We agree with the reviewer that the presented IGF2 data do not unambiguously establish IGF2 as causal downstream mediator of Endosialin. This is nowhere stated or insinuated in the manuscript. Yet, we analyzed a series of known hepatocyte mitogens including IGF1, IGF2, HGF, TGFß1, TGFß2, TGFß3, HB-EGF, FGF21, IL1P, TIMP1 and Cdkn1b. Of these, only IGF2 was significantly altered in the comparative WT vs. KO analysis. This is what we expressed in the Results section and also in the Abstract. Future work will need to unravel the detailed molecular mechanisms of Endosialin signaling that mediates autocrine effects on the pericyte/myofibroblast compartment and paracrine effects on the hepatocyte compartment.

COMMENT 6: Simply showing that recombinant IGF2 promotes proliferation of cultured hepatocytes is not sufficient to prove that increased IGF2 is the basis for the phenotype observed in the EN-KO mice.

RESPONSE: As outlined in our response to comment 5, we fully agree with the reviewer that cell culture stimulation experiments do not conclusively explain the observed phenotype. However, the aim of the stimulation experiments using recombinant IGF2 was to address reviewer 3´s comment to prove that IGF2 is not only a marker of proliferation, but truly a mitogenic factor.

COMMENT 7: Data in Fig 3j is interpreted as showing that LSECs are a key source of IGF2 in the EN-KO mice. However, the figure does not indicate whether these differences in mRNA expression are statistically significant - are they?
The text and supplemental data indicate that EN-KO HSC generate something that promotes LSECs to increase their production of IGF2. However, no experimental evidence is presented to substantiate this claim. Does immunohistochemistry demonstrate increased

IGF2 in resident LSECS of EN-KO mice? Do isolated LSECs from EN-KO mice express more IGF2 when analyzed by FACS or Western blot?

RESPONSE 7: We identified significantly elevated levels of IGF in whole liver lysates of ENKO mice. Yet, we were not able to identify one single source of IGF2. We have rephrased the text accordingly to avoid any insinuating wording. A review of the recent literature is in line with these observations: Different authors have shown that cells of mesenchymal (e.g. Hale LJ et al., J Pathol 2013; Ebisawa K et al., Biosci Bioeng 2011) as well as of epithelial origin (e.g. O´Dell SD et al., Int J Biochem Cell Biol 1998; Hashida Y et al., Minerva Gastroenterol Dietol 2011) are able to secrete IGF2. We attempted to localize IGF2 immunohistochemically. Yet, as with many secreted molecules, these experiments did not yield unambiguous results.

COMMENT 8: Does conditioned medium from EN-KO HSC stimulate LSECs from EN-KO or WT mice to produce IGF2? If direct cell-cell contact is necessary for this to occur (as the authors suggest), does conditioned medium from co-cultures of EN-KO HSC and LSECs have more IGF2 than conditioned medium from co-cultures of WT HSC and LSECs?

RESPONSE 8: The reviewer makes an excellent point. We have tried to perform such co-culture experiments, but failed because HSC and LSEC have rather different cell culture requirements. Moreover, both cell types undergo substantial phenotypic changes when transferred in culture (e.g., LSEC: closure of fenestrae, capillarization; HSC: spontaneous activation). The results of more specialized experiments, such as co-culture experiments, are consequently inherently variable and the results of such experiments may not be particularly reliable. Lastly, as we detected IGF2 in several cell types of the liver, we did not further pursue experiments on the crosstalk between HSC and LSEC.

COMMENT 9: Finally, many cell types express receptors for IGF2, including HSC themselves. Does IGF2 influence the transdifferentiation of HSC into αSMA-positive, fibrogenic myofibroblasts?

RESPONSE 9: With the primary focus of the study being on Endosialin, we did not further pursue this line of research. Clearly, as outlined above, it is intriguing that we found IGF2 rather selectively regulated and none of the other analyzed hepatotrophic cytokines. Yet, substantial additional work will be required to functionally establish IGF2 as bona fide downstream agonist of Endosialin function.

REVIEWER 3

COMMENT: All three Referees have initially agreed that the paper is lacking mechanistic insight into the shown phenotype. Reading now all referees' comments again, I would probably not have sent it out for Revision, given this fundamental critics. However, the authors have spent significant efforts on this resubmission, which is acknowledged by this Referee. I disagree with the authors that a lack of mechanistic insights justifies publication in EMM "in the Spirit of a Report". To my understanding, a Report should highlight a clear cut, less complex mechanism, rather than a pure phenotype. Also, I am not yet completely convinced on IGF2 in this process, but the authors provided some more data on this. Together, I agree with publication, mainly based on the significant efforts spent on this Revision.

RESPONSE: Following the comments of Reviewer 2 and this Reviewer, we have toned down the possible role of IGF2 as downstream regulator of Endosialin function to focus on its rather selective regulation compared to the other analyzed hepatotrophic cytokines.
We most sincerely appreciate the overall endorsement of our manuscript by this Reviewer. While the underlying molecular mechanisms have not been worked out in detail, we are confident that the data will stand the test of time and – given the presently pursued translational exploitation of Endosialin as a therapeutic target in oncological settings – we are optimistic that the work may and will stimulate further work aimed at exploiting Endosialin as therapeutic target in non-oncological settings, most notably as it relates to pathological fibrosis.
