# Supplementary figures and images for: Hepatic stellate cell-expressed endosialin balances fibrogenesis and hepatocyte proliferation during liver damage
Source: EMBO Mol Med. 2015 Feb 13;7(3):332–8. doi: 10.15252/emmm.201404246 (PMC4364949; doi:10.15252/emmm.201404246)

Fig. 2i

PDGFR $\beta$

250 —

130 —

100 —

70 —

55 —

35 —

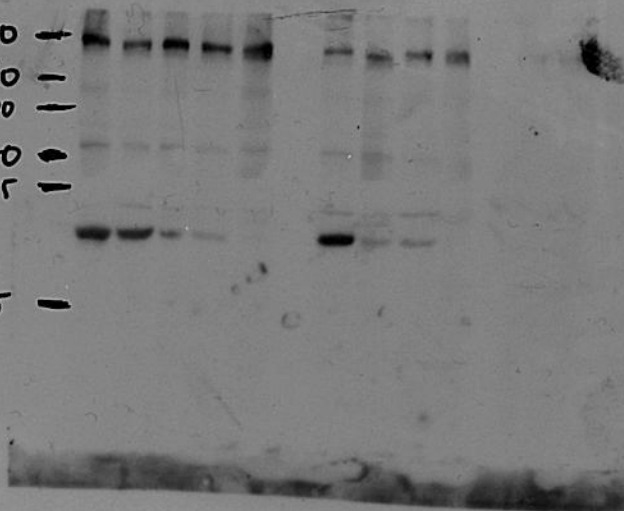

Fig. 2c

$\beta$ -actin

55 —

35 —

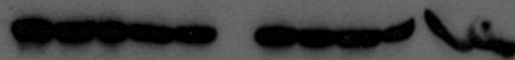

Supplement: Supplementary file 3 [file emmm0007-0332-sd3.pdf]

Fig 3h

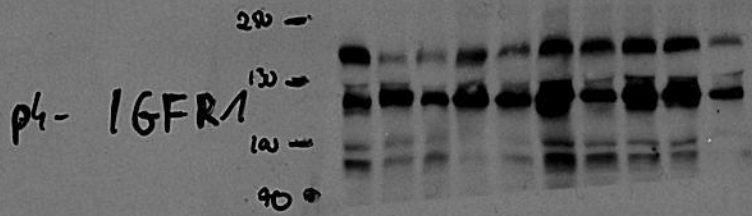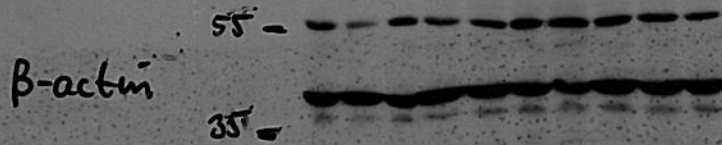

Fig 3h

p-IRS1

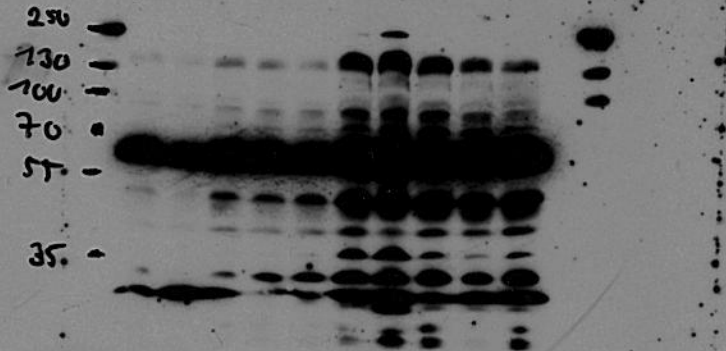

Fig. 3i

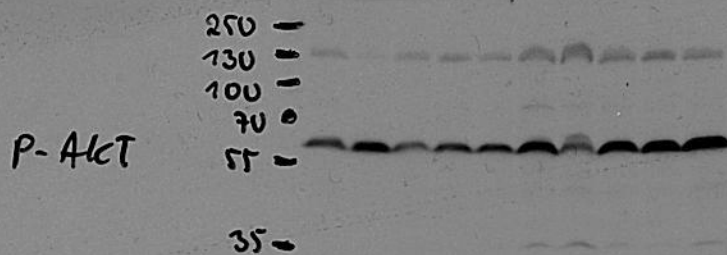

Fig 3i

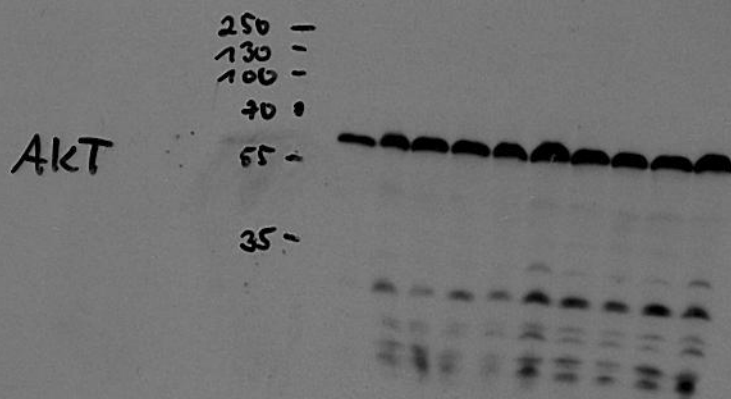

Fig 3i

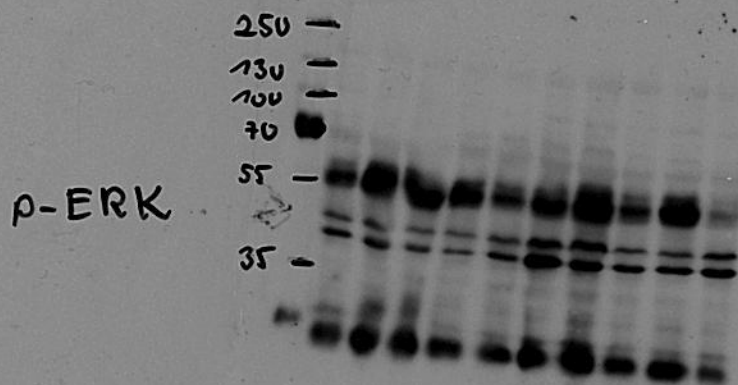

Fig 3i

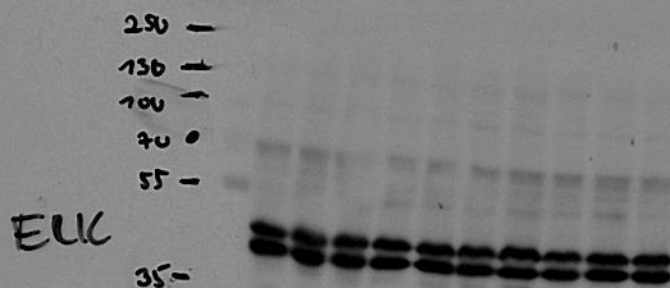

Supplement: Supplementary file 4 [file emmm0007-0332-sd4.pdf]
